# Daily injection of the β2 adrenergic agonist clenbuterol improved poor muscle growth and body composition in lambs following heat stress-induced intrauterine growth restriction

**DOI:** 10.3389/fphys.2023.1252508

**Published:** 2023-09-06

**Authors:** Rachel L. Gibbs, Rebecca M. Swanson, Joslyn K. Beard, Zena M. Hicks, Micah S. Most, Haley N. Beer, Pablo C. Grijalva, Shawna M. Clement, Eileen S. Marks-Nelson, Ty B. Schmidt, Jessica L. Petersen, Dustin T. Yates

**Affiliations:** Stress Physiology Laboratory, Department of Animal Science, University of Nebraska-Lincoln, Lincoln, NE, United States

**Keywords:** developmental origins of health and disease, DOHaD, fetal growth restriction, fetal programming, growth efficiency, low birthweight

## Abstract

**Background:** Intrauterine growth restriction (**IUGR**) is associated with reduced β2 adrenergic sensitivity, which contributes to poor postnatal muscle growth. The objective of this study was to determine if stimulating β2 adrenergic activity postnatal would rescue deficits in muscle growth, body composition, and indicators of metabolic homeostasis in IUGR offspring.

**Methods:** Time-mated ewes were housed at 40°C from day 40 to 95 of gestation to produce IUGR lambs. From birth, IUGR lambs received daily IM injections of 0.8 μg/kg clenbuterol HCl (**IUGR+CLEN**; *n* = 11) or saline placebo (**IUGR**; *n* = 12). Placebo-injected controls (*n* = 13) were born to pair-fed thermoneutral ewes. Biometrics were assessed weekly and body composition was estimated by ultrasound and bioelectrical impedance analysis (**BIA**). Lambs were necropsied at 60 days of age.

**Results:** Bodyweights were lighter (*p* ≤ 0.05) for IUGR and IUGR+CLEN lambs than for controls at birth, day 30, and day 60. Average daily gain was less (*p* ≤ 0.05) for IUGR lambs than controls and was intermediate for IUGR+CLEN lambs. At day 58, BIA-estimated whole-body fat-free mass and ultrasound-estimated loin eye area were less (*p* ≤ 0.05) for IUGR but not IUGR+CLEN lambs than for controls. At necropsy, loin eye area and *flexor digitorum superficialis* muscles were smaller (*p* ≤ 0.05) for IUGR but not IUGR+CLEN lambs than for controls. *Longissimus dorsi* protein content was less (*p* ≤ 0.05) and fat-to-protein ratio was greater (*p* ≤ 0.05) for IUGR but not IUGR+CLEN lambs than for controls. *Semitendinosus* from IUGR lambs had less (*p* ≤ 0.05) β2 adrenoreceptor content, fewer (*p* ≤ 0.05) proliferating myoblasts, tended to have fewer (*p* = 0.08) differentiated myoblasts, and had smaller (*p* ≤ 0.05) muscle fibers than controls. Proliferating myoblasts and fiber size were recovered (*p* ≤ 0.05) in IUGR+CLEN lambs compared to IUGR lambs, but β2 adrenoreceptor content and differentiated myoblasts were not recovered. *Semitendinosus* lipid droplets were smaller (*p* ≤ 0.05) in size for IUGR lambs than for controls and were further reduced (*p* ≤ 0.05) in size for IUGR+CLEN lambs.

**Conclusion:** These findings show that clenbuterol improved IUGR deficits in muscle growth and some metabolic parameters even without recovering the deficit in β2 adrenoreceptor content. We conclude that IUGR muscle remained responsive to β2 adrenergic stimulation postnatal, which may be a strategic target for improving muscle growth and body composition in IUGR-born offspring.

## 1 Introduction

Heat stress during pregnancy induces intrauterine growth restriction (IUGR) of the placenta and fetus, which results in low birthweight of the offspring (Limesand et al., 2018; Beede et al., 2019). Stress-altered fetal programming of skeletal muscle disproportionally reduces that tissue’s growth potential, which in turn diminishes growth efficiency, carcass yield, and value in livestock (Gibbs and Yates, 2021; Hicks and Yates, 2021) and contributes to metabolic health disorders in humans (Brown and Hay, 2016). Offspring born with low birthweight due to IUGR have hallmark deficiencies in lean muscle mass and greater propensity for fat deposition, which results in asymmetric body composition (Bell and Greenwood, 2016; Gibbs et al., 2019; Greenwood and Bell, 2019). As IUGR-born offspring age, these deficits expand to include poor metabolic function that makes growth less efficient in addition to being slower (Yates et al., 2018). Low birthweight due to IUGR most commonly results from placental insufficiency, which yields progressive fetal O_2_ and nutrient deficits during late gestation (Burton and Jauniaux, 2018; Limesand et al., 2018). Circulating fetal catecholamines are elevated in response to hypoxemia and hypoglycemia, which help to redirect nutrients for preferential utilization by brain, bone, and endocrine tissues that are most critical for fetal survival (Yates et al., 2011; Limesand and Rozance, 2017; Posont and Yates, 2019; Davis et al., 2020). Over time, the fetus adapts to heightened catecholamine exposure by downregulating tissue sensitivity to adrenergic stimulation. This includes reduced expression of the β2 adrenoceptor and in β2 adrenergic responsiveness (Chen et al., 2010; Yates et al., 2019; Gibbs and Yates, 2021; Cadaret et al., 2022). Because β2 adrenergic signaling stimulates muscle nutrient utilization and increases protein synthesis in skeletal muscle (Claeys et al., 1989; Anthony and Henry, 2015; Cadaret et al., 2017), reductions in β2 adrenergic activity observed in IUGR muscle pose a liability for postnatal lean muscle growth and metabolic homeostasis (Gibbs and Yates, 2021; Hostrup and Onslev, 2022). We hypothesized that targeting this loss in β2 adrenergic tone via early-life treatment with a β2 adrenergic agonist would improve dysfunctional muscle growth and metabolic homeostasis observed in IUGR-born offspring. Thus, the objective of this study was to assess the effects of stimulating β2 adrenergic activity with injectable clenbuterol from birth to weaning age on postnatal muscle growth capacity, body composition, and metabolic indicators in juvenile IUGR-born lambs.

## 2 Materials and methods

### 2.1 Animals and experimental design

These studies were approved by the Institutional Animal Care and Use Committee at the University of Nebraska-Lincoln, which is accredited by AAALAC International. Placental insufficiency-induced IUGR lambs were produced from Polypay-crossbred ewes as previously described (Beede et al., 2019; Cadaret et al., 2022). Briefly, timed-mated ewes (18–24 months of age at breeding) were exposed to ambient temperatures of 40°C + 35% relative humidity (Temperature-Humidity Index = 86) for 12 h/day and 35°C + 35% relative humidity (Temperature-Humidity Index = 82) for the remaining 12 h/day from the 40th to the 95th day of gestation, which coincided with peak placental development. On day 96 of gestation, ewes were returned to thermoneutral conditions (25°C + 15% relative humidity; Temperature-Humidity Index = 68) for the remainder of gestation. Ewes carrying control lambs were housed under thermoneutral conditions and pair-fed to the average of the ewes carrying IUGR lambs. Nutritional management, housing, and husbandry for ewes were performed as previously described (Posont et al., 2021). Lambs were separated from ewes at birth, fed a minimum of 200 mL colostrum (pooled from multiple ewes) over the first 24 h, and then reared on milk replacer (Land O’Lakes Inc., Arden Hills, MN) exclusively until 30 days of age. All lambs were fed by hand every 4 h until capable of eating from stationary bottles, typically within 5 days of birth. They were then offered *ad libitum* milk replacer from hanging bottles that were replaced twice daily. Beginning at 30 days of age, lambs were transitioned to an *ad libitum* grain diet (Supplementary Table S1), and milk replacer was discontinued at 45 days of age. At birth, IUGR-born lambs were randomly assigned to receive daily intramuscular injections of saline (IUGR; *n* = 12 from 8 ewes; 2 singletons and 10 twins; 5 males and 6 females) or 0.8 μg/kg clenbuterol HCl (IUGR+CLEN; *n* = 11 from 7 ewes; 2 singletons and 9 twins; 6 males and 5 females; MilliporeSigma, Burlington, MA, United States). Clenbuterol HCl has a circulating half-life of 9–35 h in rodents (Yamamoto et al., 1985), 18 h in calves (Sauer et al., 1995), and 35 h in goats (Zhao et al., 2015). Control lambs (*n* = 13 from 8 ewes; 2 singletons and 11 twins; 7 males and 6 females) also received daily saline injections. At 55 days of age, gas-sterilized Tygon catheters were placed in the descending aorta via the femoral artery under general anesthesia as previously described (Posont et al., 2021; Cadaret et al., 2022). Catheters were tunneled subcutaneously, exteriorized at the flank, stored in a plastic mesh pouch that was sutured to skin, and flushed twice daily with heparinized saline. Daily arterial blood samples were collected from day 56–60. Total blood volume collected did not exceed 20 mL for any 24-h period. Lambs were euthanized via barbiturate overdose and necropsied at 60 ± 1 day of age.

### 2.2 Biometrics

#### 2.2.1 Growth

Lamb bodyweights (BW) and feed intake were recorded daily. Crown circumference, front cannon bone length, abdominal circumference (i.e., girth), and crown-rump length were measured at birth and weekly thereafter. At necropsy, brain, heart, lungs, kidneys, liver, hindlimb, and *flexor digitorum superficialis* muscles were weighed. Lamb carcasses were chilled for 24 h and split between the 12th and 13th rib to measure loin-eye area (LEA), which is the cross-sectional area of the *longissimus dorsi* muscle, using the Iowa State University Plastic Grid for Quick Measurement.

#### 2.2.2 Body and muscle composition

Bioelectrical impedance analysis (**BIA**) was performed on live lambs at days 30 and 58 and on the skinned, eviscerated carcass at necropsy to estimate moisture, protein, and fat content, fat-free mass, fat-free soft tissue, the combined mass of the leg, sirloin, and loin (LSL), the leg, sirloin, loin, rack, and shoulder (LSLRS), and the leg, sirloin, rack, shoulder, neck, riblets, shank, and lean trim (SUM) as previously described (Berg and Marchello, 1994; Gibbs et al., 2019). A four-terminal Quantum V unit (RJL Systems, Detroit, MI) was used to measure reactance (Xc), resistance (Rs), and phase angle (PA). Each BIA assessment utilized two sets of equally-spaced electrode terminals to transmit an electrical current across the tissues. Electrodes were connected to aluminum 20G MONOJECT needles (Covidien, Mansfield, MA) and placed subcutaneous for live lambs and intramuscular for the *longissimus dorsi*. The outer electrodes were placed 2.5 cm behind the point of the scapula and 5 cm in front of the leading edge of the pelvis, respectively, and the inner electrodes were placed 2.5 cm inside of these. Both electrode sets were placed 2 cm to the right of the dorsal midline. Six consecutive measurements of 5 s each were recorded and averaged. At necropsy, the contralateral *longissimus dorsi* was frozen for proximate analysis to determine moisture, protein, fat, ash, carbohydrate, and caloric content (Midwest Laboratories, Omaha, NE). Ultrasonic estimates of the LEA, loin depth, and backfat thickness were performed at 58 days of age as previously described (Swanson et al., 2020). Lambs were shorn from the midline to the lower right flank. An IBEX PRO (E.I. Medical Imaging, Loveland, CO) ultrasound with an L6.2 12-cm linear transducer was used to capture images of the area between the 12th and 13th ribs. The transducer was initially placed at an ∼45° angle toward the head of the animal following heavy application of vegetable oil couplant. Measurements were determined from still images utilizing the caliper tracing mode.

### 2.3 Daily blood parameters

Daily arterial blood samples were collected into heparinized and EDTA syringes. Glucose, lactate, pH, partial pressure of CO_2_ (pCO_2_), partial pressure of O_2_ (pO_2_), HCO_3_^−^, oxyhemoglobin, carboxyhemoglobin, Na^+^, K^+^, Cl^−^, and Ca^2+^ were measured in heparinized whole blood with an ABL90 FLEX blood gas analyzer (Radiometer, Brea, CA). Total and differential white blood cell counts, hematocrit, mean corpuscular volume, red blood cell distribution width, hemoglobin, mean corpuscular hemoglobin concentration, red blood cells, platelets, and mean platelet volume were measured in EDTA-treated whole blood with a HemaTrue Veterinary Hematology Analyzer (Heska Corp., Loveland, CO). Plasma was separated from EDTA-treated whole blood via centrifuge (14,000 x *g*, 2 min) and stored at −80°C. Plasma insulin and non-esterified fatty acid (NEFA) concentrations were determined from 50-μL and 5-μL duplicate aliquots, respectively, with commercial ELISA kits (Bovine Insulin; Alpco, Windham, NE and NEFA-HR (2); Fujifilm, Richmond, VA) as previously described (Cadaret et al., 2022; King et al., 2022). Intra-assay and inter-assay coefficients of variance were less than 15% for both assays. Blood plasma urea nitrogen (BUN), triglycerides, and high-density lipoprotein-bound cholesterol (HDL-C) concentrations were determined with a Vitros 250 Chemistry Analyzer (Ortho Clinical Diagnostics, Raritan, NJ) by the University of Nebraska Biomedical and Obesity Research Core as previously described (Swanson et al., 2020). Total plasma cholesterol was also measured, but values for almost all lambs were below the detection limit of 45 mg/dL, and thus these data are not included.

### 2.4 Muscle histology and immunoblots

#### 2.4.1 Muscle fibers and myoblast populations

Muscle fiber cross-sectional areas and myoblast populations were assessed in *semitendinosus* and *longissimus dorsi* muscle samples via immunofluorescent staining as previously described (Yates et al., 2016). Myoblast populations were also assessed in *biceps femoris* muscle samples. Briefly, cross-sectional samples from the belly of each muscle were collected at necropsy and fixed with 4% paraformaldehyde in phosphate-buffered saline (PBS). Samples were then embedded in OCT compound and stored at −80°C. Cryosections (10 µm) were mounted on charged microscope slides (Thermo Fisher Scientific, Waltham, MA, United States) and dried for 2 h at 37°C. Slides were then rehydrated with PBS and antigen retrieval was performed by boiling and cooling in 10 mM citric acid. Non-specific staining was blocked by incubating slides in 0.5% NEN blocking buffer (PerkinElmer, Inc., Waltham, MA) in PBS for 1 h in a humidified container at room temperature. Slides were then incubated overnight at 4°C with primary antibodies diluted in PBS + 1% bovine serum albumin (MilliporeSigma). Negative controls were incubated in PBS without primary antibody. Muscle sections were stained with mouse antiserum raised against pax7 (1:10; Developmental Studies Hybridoma Bank (DSHB), Iowa City, IA, United States) to identify myoblasts and counterstained with rabbit antiserum raised against proliferating cell nuclear antigen (PCNA; 1:10; DSHB) to identify cells undergoing proliferation. Additional sections were stained with mouse antiserum raised against myogenin (1:5; DSHB) to identify differentiated myoblasts and counterstained with rabbit antiserum raised against desmin (1:300; MilliporeSigma) to assess muscle fiber area. Immunocomplexes were detected with affinity-purified immunoglobulin antiserum conjugated to AlexaFluor 488, AlexaFluor 555, or AlexaFluor 594 (1:1,000; Cell Signaling Technologies, Danvers, MA, United States). Immunofluorescent images were visualized on an Olympus IX73 and digitally captured with a DP80 microscope camera (Olympus Corp., Center Valley, PA, United States). Images were analyzed with Olympus cellsSens Dimension software to determine myoblast population profiles and muscle fiber area. Animal identifications and experimental designations were deidentified prior to analyses. Myoblast populations within each muscle were assessed from a minimum of 800 nuclei across 3 non-overlapping fields of view. Average fiber area for each muscle was determined from a minimum of 100 muscle fibers across 3 non-overlapping fields of view.

#### 2.4.2 Lipid droplet profiles

Cross-sectional samples of the *semitendinosus* were analyzed for mean intramuscular lipid droplet size and for size distributions. Briefly, samples collected at necropsy were fixed with 4% paraformaldehyde, embedded in OCT compound, and stored at −80°C. Cryosections (10 µm) were mounted on charged microscope slides. Slides were brought to room temperature, rinsed in 60% isopropanol, and incubated with Oil Red O (MilliporeSigma) working solution for 15 min. Sections were then washed with 60% isopropanol and de-ionized water before mounting with hydromount (National Diagnostics, Atlanta, GA, United States). Images were visualized and captured as described above, and mean lipid droplet area and lipid size distributions were determined with ImageJ Software across 6 non-overlapping fields of view.

#### 2.4.3 Protein immunoblots

Total protein was isolated from *semitendinosus* that was snap-frozen in liquid nitrogen at necropsy and used to determine β2 adrenoceptor (Adrβ2) protein content, as previously described (Yates et al., 2019; Cadaret et al., 2022). Briefly, muscle samples were homogenized via sonication (3 × 5 s) in RIPA buffer containing 2.5% protease and 2.5% phosphatase inhibitor and then centrifuged (14,000 x *g*, 5 min, 4°C). Total protein concentrations were quantified from the supernatant utilizing a Piece BCA Assay Kit (Thermo Fisher). A 40-μg protein aliquot was then mixed with Bio-Rad 4x Laemmli sample buffer (Bio-Rad Laboratories, Hercules, CA) and heated at 95°C for 5 min. Samples were allowed to cool to room temperature before being separated by SDS-PAGE and transferred to poly-vinylidene fluoride low-fluorescent membranes (Bio-Rad). Membranes were incubated in Bio-Rad EveryBlot Blocking Buffer for 10 min at room temperature and washed with TBS-T prior to primary antibody incubation. Membranes were incubated with rabbit anti-serum raised against Adrβ2 (1:1000, Cohesion Biosciences, London, United Kingdom) overnight at 4°C. Membranes were then washed with TBS-T and incubated with goat anti-rabbit IR800 IgG secondary anti-serum (LI-COR Biosciences, Lincoln, NE, United States) for 1 h at room temperature. Membranes were scanned with the Odyssey Infrared Imaging System and analyzed with Image Studio Lite Software Ver 5.2 (LI-COR). This resulted in quantification of total cellular content of Adrβ2 and did not distinguish between surface membrane and sequestered fractions.

### 2.5 Statistical analysis

Immunohistochemistry and other data collected at necropsy were analyzed by ANOVA using the mixed procedure of SAS 9.4 (SAS Institute, Cary, NC, United States) for the fixed effects of experimental group, sex, and birth number. Interactions among these effects were not included due to insufficient power. However, all sex and birth number categories were represented in all groups as reported in the methods. Fisher’s LSD test was used for mean separation. Daily/weekly growth metrics and blood components were analyzed using the mixed procedure with repeated measures to analyze the effects of experimental group, age in days, and group × age interaction, as well as sex and birth number. Best-fit statistics were used to select appropriate covariance structures. Placental anastomosis is rare in sheep (Dain, 1971) and placental effects from experimental conditions were assumed to be distinct for each fetus. Therefore, lamb was considered the experimental unit. Significant differences for all analyses were identified by a *p*-value of ≤ 0.05, and tendencies toward differences were indicated by *p*-values of ≤ 0.10. All data are presented as least-squares means ± standard error of the mean. Potential limitations for this study may include the grouping of males and females, the use of singleton and twin-born lambs, the assumed independence of placental effects between twins, and the use of pooled colostrum.

## 3 Results

### 3.1 Growth metrics

**FIGURE 1:**
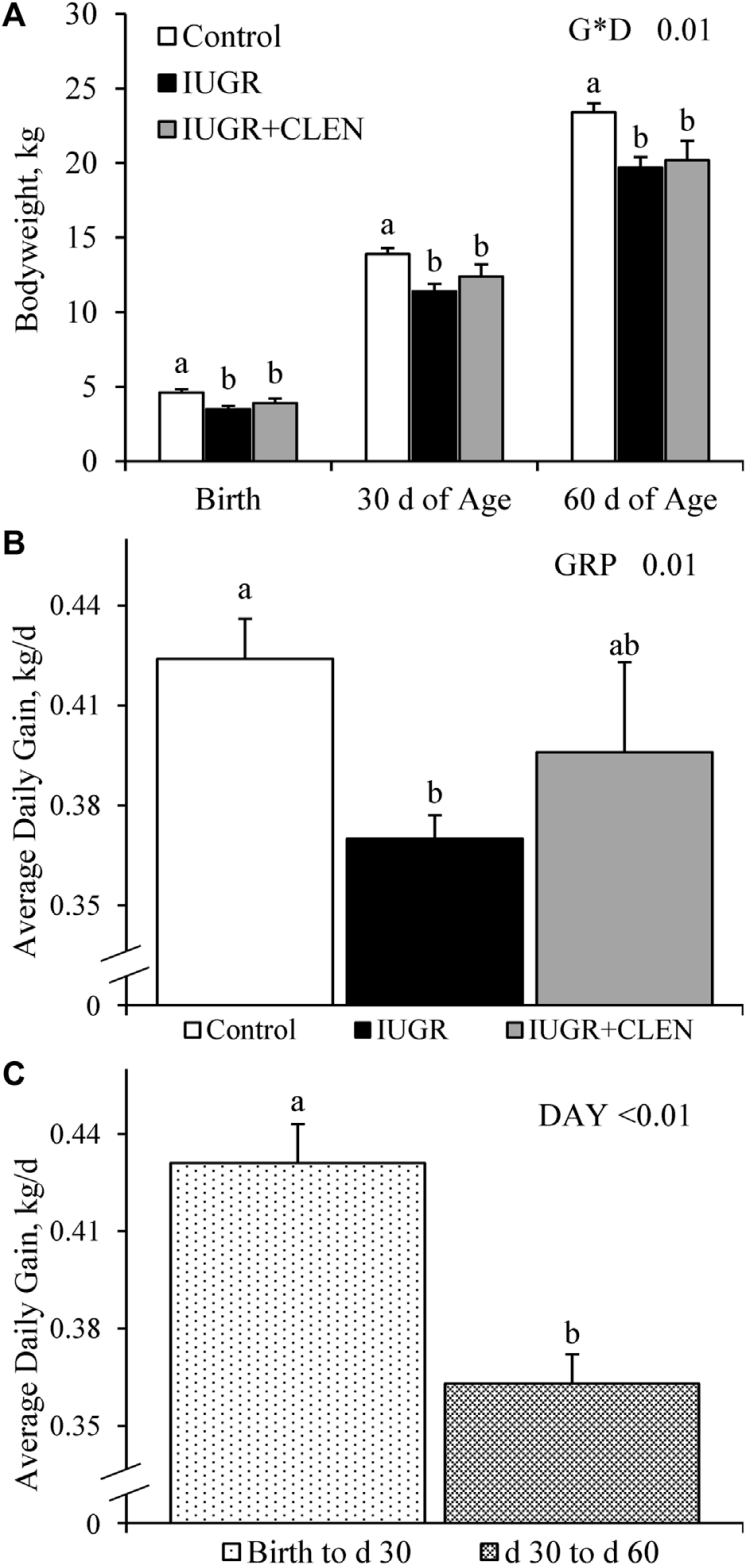
Growth in IUGR-born lambs administered daily injectable clenbuterol. Fasted weights were assessed in controls (*n* = 13), IUGR lambs (*n* = 12), and IUGR+CLEN lambs (*n* = 11) at birth, 30 days of age, and 60 days of age. Data are presented for bodyweight **(A)**, average daily gain from birth to 60 days of age for each experimental group **(B)**, and average daily gain from birth to 30 days of age and 30–60 days of age for all lambs **(C)**. Effects of experimental group (GRP), day (DAY), and the interaction (G*D) were evaluated and noted where significant (*p* < 0.05). ^a, b^ Means with different superscripts differ (*p* < 0.05).

An experimental group × age interaction was observed (*p* ≤ 0.05) for BW, but not for average daily gain, crown circumference, abdominal circumference, crown-rump length (i.e., body length, BL), or cannon bone length. Respectively, IUGR and IUGR+CLEN lambs weighed less (*p* ≤ 0.05) than controls by 24% and 15%, at birth, by 18% and 11%, at 30 days of age, and by 16% and 13% at 60 days of age (Figure 1A). Daily BW are presented in Supplementary Figure S1. Average daily gain from birth to 60 days of age was reduced (*p* ≤ 0.05) for IUGR lambs compared to controls and was intermediate for IUGR+CLEN lambs (Figure 1B). Moreover, average daily gain from birth to 30 days of age was greater (*p* ≤ 0.05) than from 30 to 60 days for all lambs, regardless of experimental group (Figure 1C). Crown circumference, abdominal circumference, and crown-rump length were less (*p* ≤ 0.05) for IUGR and IUGR+CLEN than for controls, regardless of age (Supplementary Table S2). Crown circumference/BW, abdominal circumference/BW, crown-rump length/BW, cannon bone length/BW, and cannon bone length/BL were greater (*p* ≤ 0.05) and cannon bone length/abdominal circumference tended to be greater (*p* = 0.06) for IUGR and IUGR+CLEN lambs than controls. BW, crown circumference, abdominal circumference, and crown-rump length were greater (*p* ≤ 0.05) for singletons than twin-born lambs, but no growth metrics differed between sexes.

At necropsy, whole hindlimbs tended to be lighter (*p* = 0.10) for IUGR and IUGR+CLEN lambs than for controls, but hindlimb/BW and hindlimb/BL did not differ among experimental groups (Table 1). *Flexor digitorum superficialis* (**FDS**) muscle weights and FDS/BL were less (*p* ≤ 0.05) for IUGR lambs than for controls and were intermediate for IUGR+CLEN lambs. FDS/BW did not differ among groups. Heart, lungs, liver, and kidneys were lighter (*p* ≤ 0.05) for IUGR and IUGR+CLEN lambs than for controls. Heart/BW was less (*p* ≤ 0.05) for IUGR lambs and was intermediate for IUGR+CLEN lambs. Heart/BL, lungs/BW, lungs/BL, liver/BL, kidneys/BW, and kidneys/BL were less (*p* ≤ 0.05) for IUGR and IUGR+CLEN lambs than for controls. Liver/BW did not differ between IUGR lambs and controls but was less (*p* ≤ 0.05) for IUGR+CLEN than controls. Brain weight, brain/BW, and brain/BL did not differ among experimental groups. Hindlimb, FDS, lungs, liver, and brain weights as well as hindlimb/BL, FDS/BW, and FDS/BL were greater (*p* ≤ 0.05) for singletons than twin-born lambs, but no biometrics differed between sexes.

**TABLE 1:** Absolute and relative organ mass at 60 days of age in IUGR-born lambs administered daily injectable clenbuterol.

Organ	Experimental group			*p-*value	
	Control	IUGR	IUGR+CLEN^d^		
Mass, g					
Brain	89.5 ± 4.3	86.9 ± 3.5	86.5 ± 4.4	NS	
Heart	171 ± 8^a^	138 ± 10^b^	133 ± 11^b^	0.002	
Liver	602 ± 42^a^	502 ± 35^b^	460 ± 48^b^	0.004	
Lungs	432 ± 29^a^	303 ± 20^b^	315 ± 40^b^	< 0.001	
Kidneys	64.2 ± 3.3^a^	49.2 ± 2.1^b^	45.3 ± 2.4^b^	< 0.001	
Hindlimb	2037 ± 189^x^	1846 ± 177^y^	1825 ± 223^y^	0.10	
FDS Muscle	24.6 ± 2.3^a^	21.0 ± 1.2^b^	21.4 ± 2.3^ab^	0.05	
Mass/BW, g/kg					
Brain	3.94 ± 0.14	4.2 ± 0.13	4.44 ± 0.23	NS	
Heart	7.21 ± 0.36^a^	5.51 ± 0.21^b^	6.55 ± 0.24^c^	< 0.001	
Liver	25.2 ± 0.70^a^	23.9 ± 1.30^ab^	22.0 ± 0.90^b^	0.03	
Lungs	19.07 ± 0.99^a^	15.13 ± 0.78^b^	16.17 ± 1.15^b^	0.01	
Kidneys	2.72 ± 0.07^a^	2.67 ± 0.15^a^	2.17 ± 0.23^b^	0.004	
Hindlimb	86.8 ± 2.80	88.1 ± 2.70	88.8 ± 2.80	NS	
FDS Muscle	1.05 ± 0.03	1.01 ± 0.08	1.03 ± 0.10	NS	
Mass/BL, g/cm					
Brain	0.86 ± 0.04	0.84 ± 0.03	0.86 ± 0.41	NS	
Heart	1.64 ± 0.10^a^	1.19 ± 0.06^b^	1.31 ± 0.10^b^	0.004	
Liver	5.70 ± 0.25^a^	4.83 ± 0.24^b^	4.52 ± 0.36^b^	0.01	
Lungs	4.15 ± 0.29^a^	2.99 ± 0.20^b^	3.19 ± 0.38^b^	0.001	
Kidneys	0.62 ± 0.04^a^	0.46 ± 0.01b	0.45 ± 0.02^b^	0.002	
Hindlimb	19.3 ± 0.60	17.8 ± 0.70	18.1 ± 1.20	NS	
FDS Muscle	0.24 ± 0.01^a^	0.19 ± 0.01^b^	0.21 ± 0.01^ab^	0.02	

^a, b, c^ Means with different superscripts differ (*p* ≤ 0.05).

^d^ Daily treatment with 0.8 μg/kg injectable (IM) clenbuterol HCl.

BL, body length (i.e., crown-rump length); BW, bodyweight; FDS, *flexor digitorum superficialis*; IUGR, intrauterine growth restriction; NS, not significant.

### 3.2 Live-animal body composition estimates

#### 3.2.1 Bioelectrical impedance analysis

**FIGURE 2:**
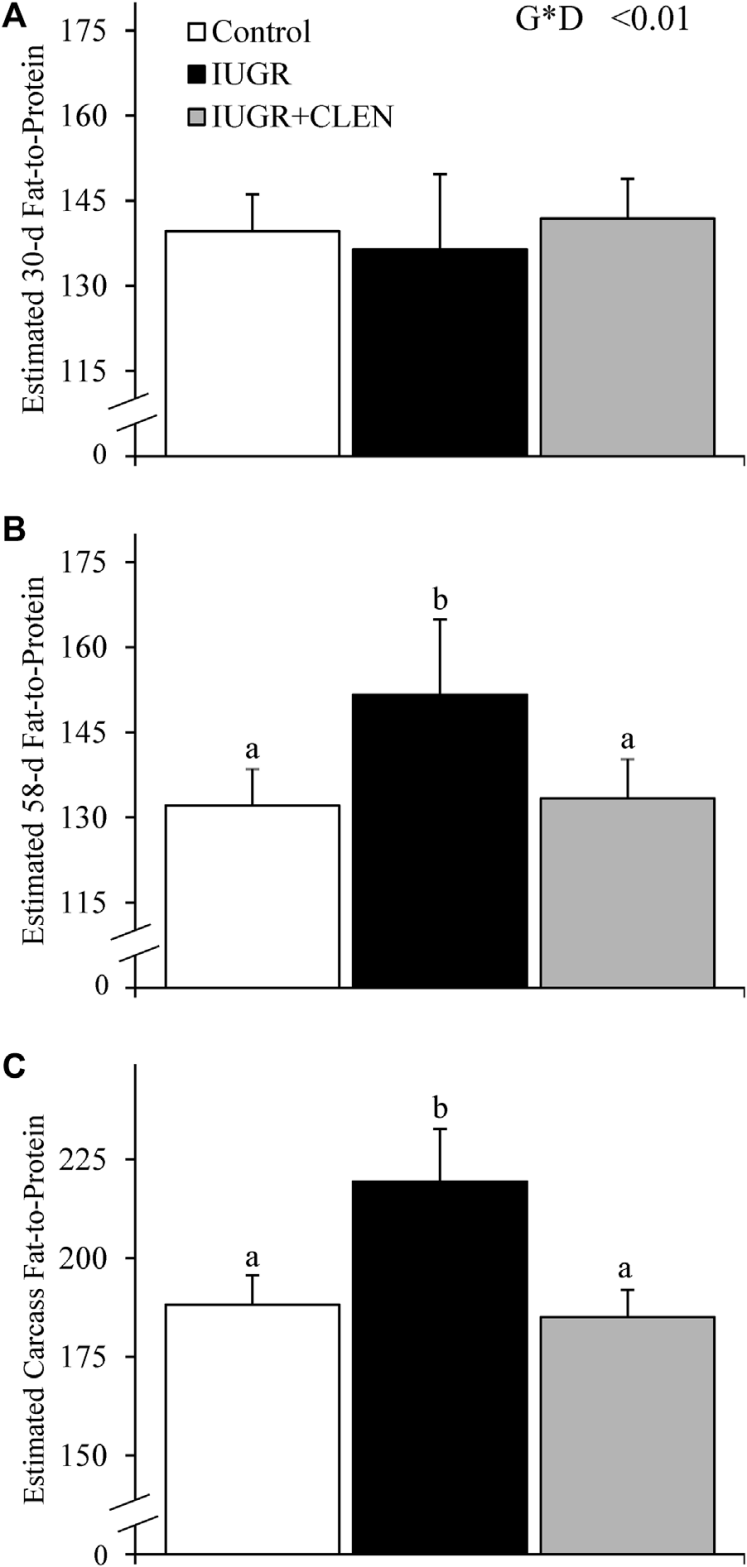
Estimated whole-body fat-to-protein ratios for IUGR-born lambs administered daily injectable clenbuterol. Bioelectrical impedance analysis (BIA) was performed in controls (*n* = 13), IUGR lambs (*n* = 12), and IUGR+CLEN lambs (*n* = 11). Data are presented for BIA-estimated fat-to-protein ratios in the live lambs at 30 days **(A)** and 58 days of age **(B)** and the carcass following necropsy at 60 days of age **(C)**. Effects of experimental group (GRP), day (DAY), and the interaction (G*D) were evaluated and noted where significant (*p* < 0.05). ^a, b^ Means with different superscripts differ (*p* < 0.05).

Experimental group × age interactions were observed (*p* ≤ 0.05) for all BIA estimations of body composition and muscle mass. At 30 days of age, BIA-estimated fat-free mass (Table 2), fat-free soft tissue, and fat-to-protein ratio (Figure 2A) did not differ among groups. However, BIA-estimated mass for SUM and LSRLS muscle groups were lighter (*p* ≤ 0.05) for IUGR lambs than for controls and were intermediate for IUGR+CLEN lambs. BIA-estimated mass for the LSL muscle group and estimates for whole-body moisture, protein, fat, and lean content did not differ among experimental groups at 30 days of age. At 58 days of age, BIA-estimated fat-free mass and fat-free soft tissue were less (*p* ≤ 0.05) for IUGR lambs than for controls. Estimated fat-free mass was recovered (*p* ≤ 0.05) in IUGR+CLEN lambs, but estimated fat-free soft tissue did not differ between IUGR and IUGR+CLEN lambs. BIA-estimated mass for SUM, LSLRS, and LSL muscle groups at days 58 were reduced (*p* ≤ 0.05) for IUGR lambs compared to controls and were intermediate for IUGR+CLEN lambs. Likewise, BIA-estimated whole-body moisture, protein, fat, and lean content were less (*p* ≤ 0.05) for IUGR lambs than controls and were intermediate for IUGR+CLEN lambs. BIA-estimated fat-to-protein ratio was greater (*p* ≤ 0.05) for IUGR lambs but not IUGR+CLEN lambs than for controls at days 58 (Figure 2B). All BIA-estimated masses were greater (*p* ≤ 0.05) for singletons than for twin-born lambs, but no estimates differed between sexes.

**TABLE 2:** Live-animal body composition estimated at 30 and 58 days of age via bioelectrical impedance analysis (BIA) in IUGR-born lambs administered daily injectable clenbuterol.

Variable	Experimental group			*p-*value
	Control	IUGR	IUGR+CLEN^c^	
30 days of age				
Fat-Free Mass, kg	8.84 ± 1.11	6.82 ± 2.21	6.68 ± 1.05	NS
Fat-Free Soft Tissue, kg	9.73 ± 1.07	7.82 ± 2.00	7.47 ± 1.08	NS
SUM, kg	7.42 ± 0.75^a^	5.97 ± 0.76^b^	5.83 ± 1.08^ab^	< 0.001
LSLRS, kg	5.20 ± 0.52^a^	4.19 ± 0.55^b^	4.09 ± 0.73^ab^	< 0.001
LSL, kg	2.75 ± 0.34	2.13 ± 0.36	2.04 ± 0.45	NS
Moisture, kg	10.17 ± 0.83	9.34 ± 1.05	8.91 ± 0.89	NS
Protein, kg	2.98 ± 0.29	2.63 ± 0.36	2.62 ± 0.29	NS
Fat, kg	2.22 ± 0.36	1.84 ± 0.47	1.63 ± 0.39	NS
Lean, kg	11.97 ± 1.09	10.60 ± 1.33	10.68 ± 1.05	NS
*58 days of age*				
Fat-Free Mass, kg	15.52 ± 1.11^a^	10.48 ± 2.21^b^	13.30 ± 1.05^a^	< 0.001
Fat-Free Soft Tissue, kg	16.00 ± 1.07^a^	11.21 ± 2.00^b^	13.69 ± 1.08^b^	< 0.001
SUM, kg	11.50 ± 0.75^a^	9.17 ± 0.76^b^	9.80 ± 1.08^ab^	< 0.001
LSLRS, kg	8.01 ± 0.52^a^	6.35 ± 0.55^b^	6.82 ± 0.73^ab^	< 0.001
LSL, kg	4.59 ± 0.34^a^	3.45 ± 0.36^b^	3.83 ± 0.45^ab^	< 0.001
Moisture, kg	14.86 ± 0.83^a^	12.24 ± 1.05^b^	13.85 ± 0.89^ab^	< 0.001
Protein, kg	4.59 ± 0.29^a^	3.80 ± 0.36^b^	4.30 ± 0.29^ab^	< 0.001
Fat, kg	4.38 ± 0.36^a^	3.18 ± 0.47^b^	3.88 ± 0.39^ab^	< 0.001
Lean, kg	17.88 ± 1.09^a^	15.06 ± 1.33^b^	16.86 ± 1.05^ab^	< 0.001

^a, b^ Means with different superscripts differ (*p* ≤ 0.05).

^c^ Daily treatment with 0.8 μg/kg injectable (IM) clenbuterol HCl.

SUM, sum of leg, sirloin, rack, shoulder, neck, riblets, shank, and lean trim; LSLRS, sum of leg, sirloin, loin, rack, and shoulder; LSL, sum of leg, sirloin, and loin; IUGR, intrauterine growth restriction; NS, not significant.

#### 3.2.2 Ultrasonography

**FIGURE 3:**
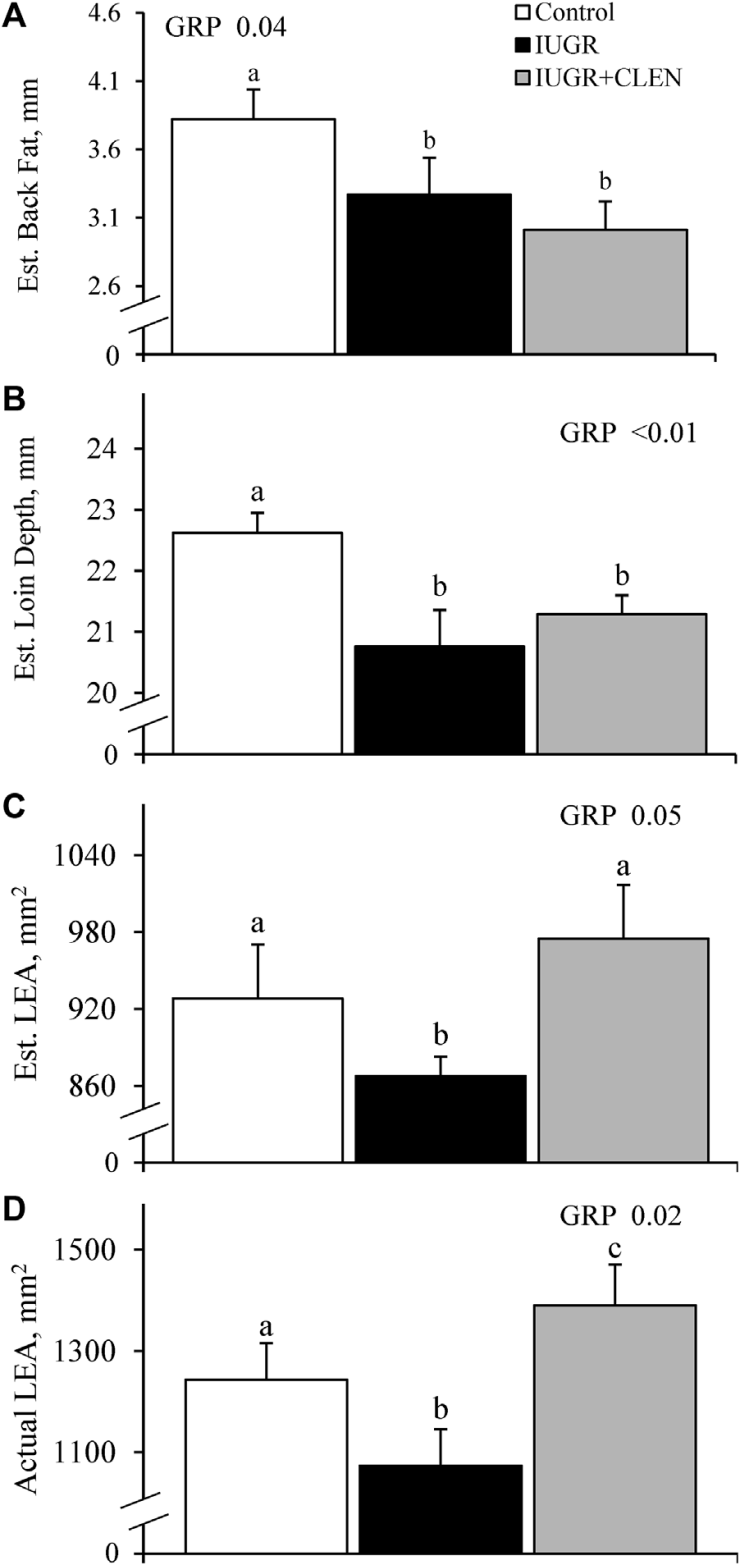
Estimated carcass traits for IUGR-born lambs administered daily injectable clenbuterol. Ultrasonic measurements were performed in controls (*n* = 13), IUGR lambs (*n* = 12), and IUGR+CLEN lambs (*n* = 11) at 58 days of age. Data are presented for estimated back fat thickness **(A)**, estimated loin depth **(B)**, and estimated loin-eye area **(C)** as well as actual loin-eye area measured in the chilled carcass **(D)** following necropsy at 60 days of age. Effects of experimental group (GRP), day (DAY), and the interaction (G*D) were evaluated and noted where significant (*p* < 0.05). ^a, b, c^ Means with different superscripts differ (*p* < 0.05).

At day 58, ultrasound-estimated back fat thickness and loin depth between the 12th and 13th ribs were less (*p* ≤ 0.05) for IUGR and IUGR+CLEN lambs than for controls (Figures 3A, B, respectively). Ultrasound-estimated LEA was less (*p* ≤ 0.05) for IUGR lambs but not IUGR+CLEN lambs than for controls (Figure 3C). Across all groups, measurements for LEA estimated by ultrasound in live animals on day 58 were moderately correlated (Pearson, r = 0.36, *p* ≤ 0.05; Spearman, r = 0.49, *p* ≤ 0.05) with actual LEA measured 24 h *postmortem* in chilled carcasses. Ultrasound-estimated back fat thickness and loin-eye depth were greater (*p* ≤ 0.05) in singletons than twin-born lambs but none of these measurements differed between sexes.

### 3.3 Carcass composition

#### 3.3.1 Bioelectrical impedance analysis

At necropsy, BIA-estimated carcass fat-free mass was less (*p* ≤ 0.05) for IUGR lambs than for controls and was intermediate for IUGR+CLEN lambs (Table 3). BIA-estimated carcass fat-free soft tissue, mass for SUM muscle groups, and mass for LSRLS muscle groups were less (*p* ≤ 0.05) for IUGR and IUGR+CLEN lambs than for controls. BIA-estimated mass for the LSL muscle group, carcass moisture content, and carcass fat content were less (*p* ≤ 0.05) for IUGR lambs than for controls and were intermediate for IUGR+CLEN lambs. Estimates for carcass protein content and lean mass were less (*p* ≤ 0.05) for IUGR lambs than for controls and IUGR+CLEN lambs. Moreover, estimated carcass fat-to-protein ratio was greater (*p* ≤ 0.05) for IUGR lambs but not IUGR+CLEN lambs than for controls (Figure 2C). All carcass estimates were greater (*p* ≤ 0.05) for singleton lambs than for twin-born lambs but did not differ between sexes.

**TABLE 3:** Postmortem carcass composition estimated at 60 days of age via bioelectrical impedance analysis (BIA) in IUGR-born lambs administered daily injectable clenbuterol.

Variable	Experimental group			*p-*value
	Control	IUGR	IUGR+CLEN^a^	
Fat-Free Mass, kg	20.65 ± 1.29^a^	16.41 ± 2.21^b^	18.65 ± 1.05^ab^	< 0.001
Fat-Free Soft Tissue, kg	23.85 ± 1.22^a^	19.63 ± 2.00^b^	21.73 ± 1.08^b^	< 0.001
SUM, kg	24.88 ± 0.77^a^	21.69 ± 0.76^b^	23.02 ± 1.08^b^	< 0.001
LSLRS, kg	16.03 ± 0.54^a^	13.84 ± 0.55^b^	14.73 ± 0.73^b^	< 0.001
LSL, kg	9.92 ± 0.36^a^	8.57 ± 0.36^b^	9.10 ± 0.45^ab^	< 0.001
Moisture, kg	11.99 ± 0.93^a^	9.42 ± 1.05^b^	10.88 ± 0.89^ab^	< 0.001
Protein, kg	3.93 ± 0.33^a^	2.97 ± 0.36^b^	3.58 ± 0.29^a^	< 0.001
Fat, kg	2.92 ± 0.40^a^	1.83 ± 0.47^b^	2.39 ± 0.39^ab^	< 0.001
Lean, kg	15.80 ± 1.21^a^	12.18 ± 1.33^b^	14.56 ± 1.05^a^	< 0.001

^a, b^ Means with different superscripts differ (*p* ≤ 0.05).

^c^ Daily treatment with 0.8 μg/kg injectable (IM) clenbuterol HCl.

SUM, sum of leg, sirloin, rack, shoulder, neck, riblets, shank, and lean trim; LSLRS, sum of leg, sirloin, loin, rack, and shoulder; LSL, sum of leg, sirloin, and loin; IUGR, intrauterine growth restriction; NS, not significant.

#### 3.3.2 Loin eye area and muscle proximate analysis

Actual LEA measured in chilled carcasses were smaller (*p* ≤ 0.05) for IUGR lambs and larger (*p* ≤ 0.05) for IUGR+CLEN lambs than for controls (Figure 3D). Proximate analysis performed on the *longissimus dorsi* indicated that muscle moisture, ash, and caloric content did not differ among experimental groups at necropsy (Supplementary Figures S2A–C, respectively). Muscle protein content was less (*p* ≤ 0.05) for IUGR but not IUGR+CLEN lambs than for controls (Supplementary Figure S2D). Muscle fat content tended to be greater (*p* = 0.08) and fat-to-protein ratio was greater (*p* ≤ 0.05) for IUGR but not IUGR+CLEN lambs compared to controls (Supplementary Figures S2E, F, respectively). Muscle carbohydrate content was greater (*p* ≤ 0.05) for IUGR and IUGR+CLEN lambs than for controls (Supplementary Figure S2G). Muscle from males had greater (*p* ≤ 0.05) moisture, ash, and caloric content but less (*p* ≤ 0.05) fat content and fat-to-protein ratio than from females but no differences were observed between singletons and twins.

### 3.4 Blood parameters

#### 3.4.1 Hematology

No experimental group × day interactions were observed for any hematological components assessed in this study. Circulating concentrations of total or differential white blood cells (lymphocytes, granulocytes, and monocytes; Supplementary Figure S3A), red blood cells (Supplementary Figure S3B), and platelets (Supplementary Figure S3C) did not differ among experimental groups. Likewise, mean corpuscular volume (32.3 ± 1.4 fL) and mean corpuscular hemoglobin concentration (39.3 ± 0.4 g/dL) did not differ among experimental groups. Hematocrit and hemoglobin concentrations did not differ between IUGR lambs and controls but were less (*p* ≤ 0.05) for IUGR+CLEN lambs (Supplementary Figures S4A, B, respectively). Red blood cell distribution widths did not differ between IUGR lambs and controls but were greater for IUGR+CLEN lambs (Supplementary Figure S4C). Mean platelet volume was greater (*p* ≤ 0.05) for IUGR lambs but less (*p* ≤ 0.05) for IUGR+CLEN lambs compared to controls (Supplementary Figure S4D). Mean platelet volume was greater (*p* ≤ 0.05) for singletons than for twin-born lambs, but no hematology parameters assessed in this study differed between sexes.

#### 3.4.2 Blood gases and metabolites

**FIGURE 4:**
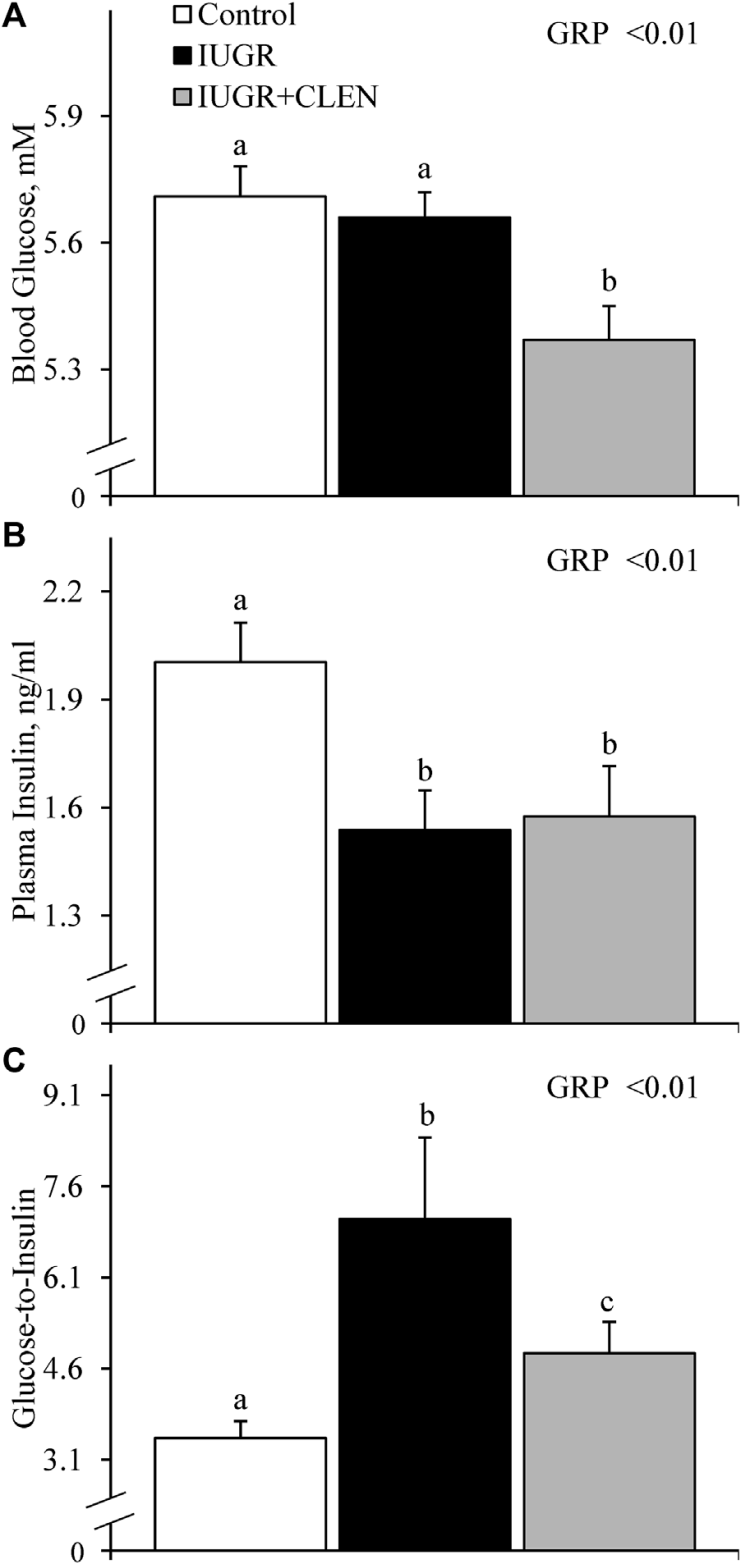
Circulating glucose and insulin for IUGR-born lambs administered daily injectable clenbuterol. Arterial blood samples were collected from controls (*n* = 13), IUGR lambs (*n* = 12), and IUGR+CLEN lambs (*n* = 11) from the 55th to 60th day of age. Data are presented for mean daily blood glucose **(A)**, mean daily plasma insulin **(B)**, and mean daily glucose-to-insulin ratios **(C)**. Effects of experimental group (GRP), day (DAY), and the interaction (G*D) were evaluated and noted where significant (*p* < 0.05). ^a, b, c^ Means with different superscripts differ (*p* < 0.05).

**FIGURE 5:**
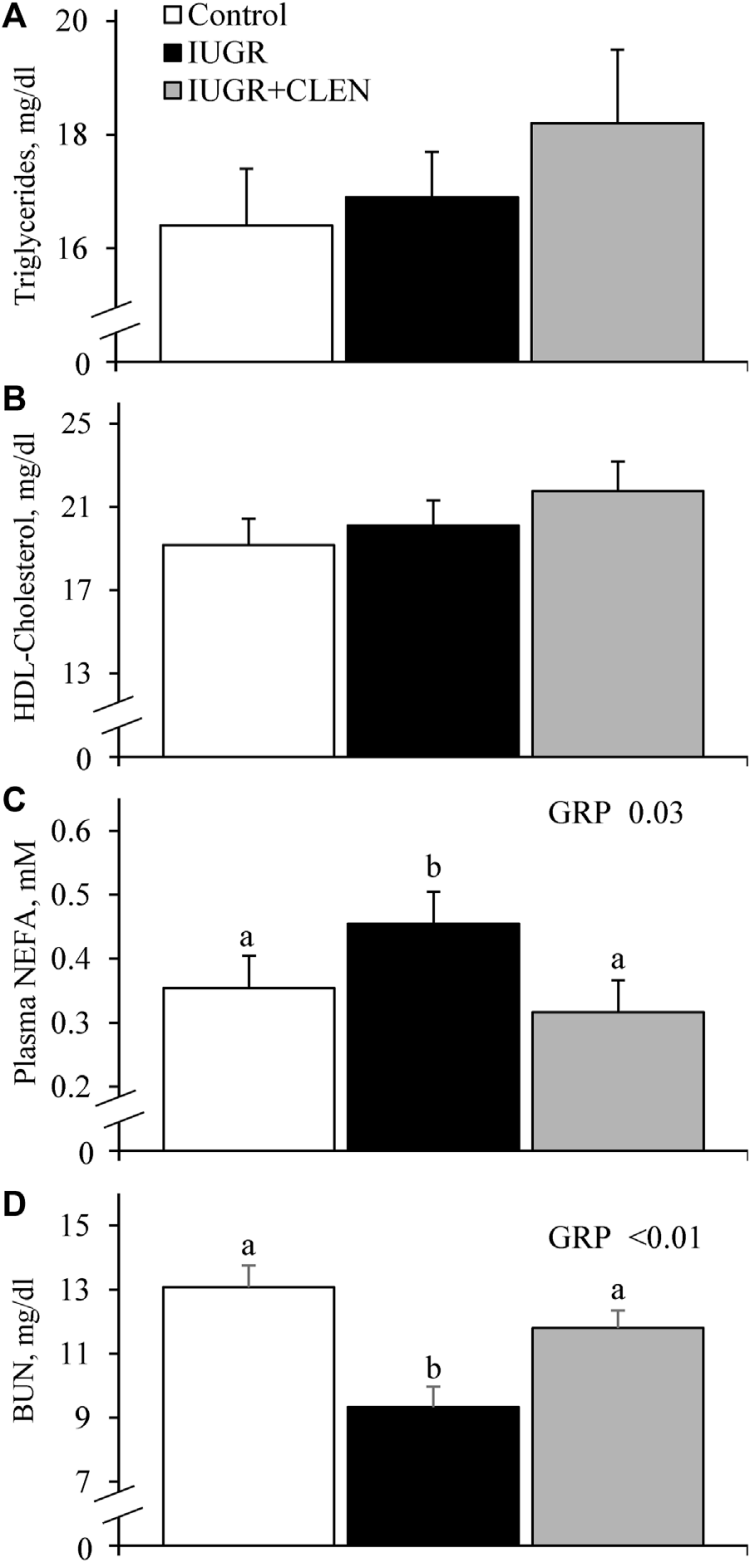
Blood metabolites for IUGR-born lambs administered daily injectable clenbuterol. Arterial blood samples were collected from controls (*n* = 13), IUGR lambs (*n* = 12), and IUGR+CLEN lambs (*n* = 11) from the 55th to 60th day of age. Data are presented for mean daily plasma triglycerides **(A)**, plasma HDL-C **(B)**, plasma non-esterified fatty acids (NEFA) **(C)** and blood plasma urea nitrogen (BUN) concentrations **(D)**. Effects of experimental group (GRP), day (DAY), and the interaction (G*D) were evaluated and are noted where significant (*p* < 0.05). ^a, b, c^ Means with different superscripts differ (*p* < 0.05).

**FIGURE 6:**
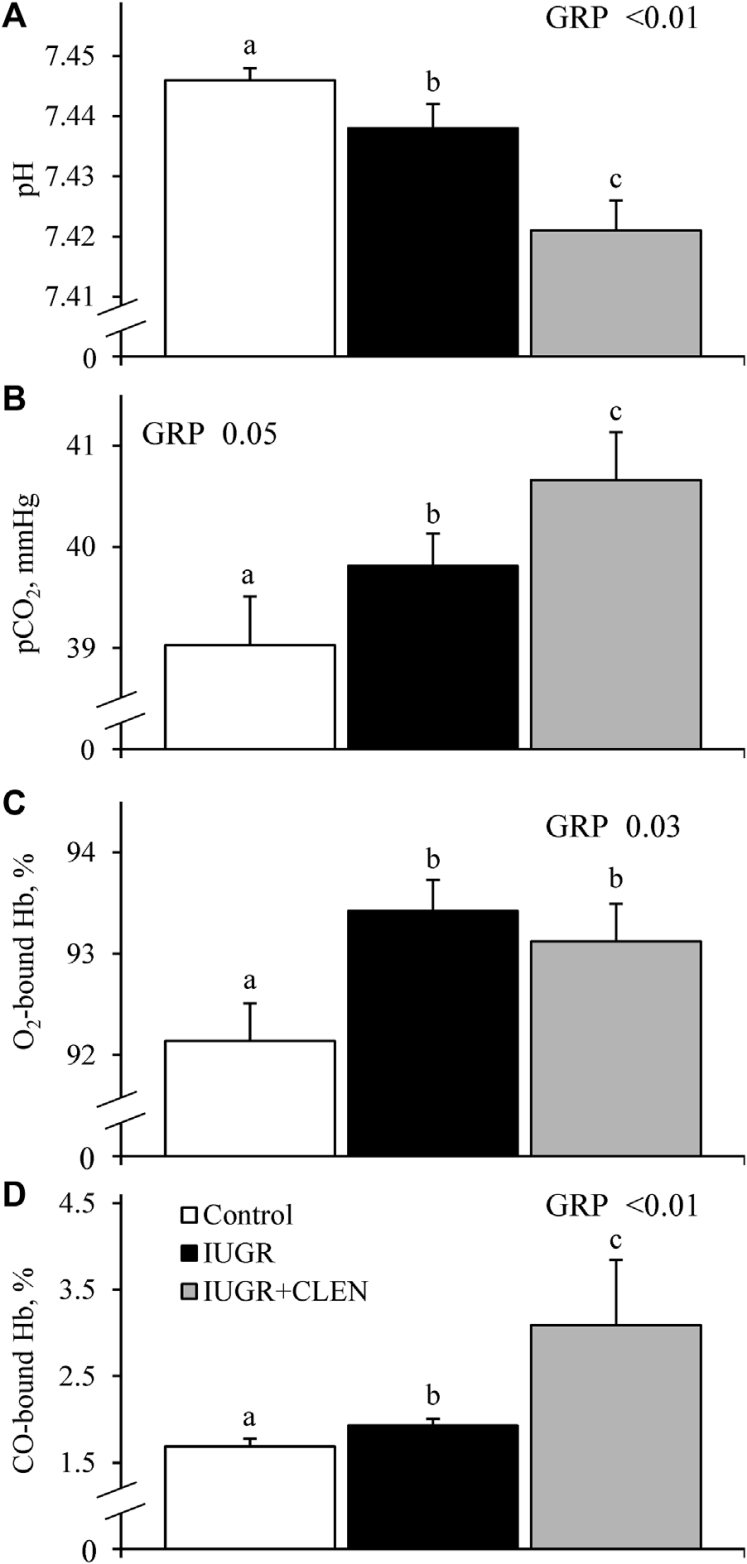
Blood gas parameters for IUGR-born lambs administered daily injectable clenbuterol. Arterial blood samples were collected from controls (*n* = 13), IUGR lambs (*n* = 12), and IUGR+CLEN lambs (*n* = 11) from the 55th to 60th day of age. Data are presented for mean daily blood pH **(A)**, blood partial pressure of CO_2_ **(B)**, blood oxyhemoglobin concentrations **(C)** and blood carboxyhemoglobin concentrations **(D)**. Effects of experimental group (GRP), day (DAY), and the interaction (G*D) were evaluated and are noted where significant (*p* < 0.05). ^a, b, c^ Means with different superscripts differ (*p* < 0.05).

No experimental group × day interactions were observed for any blood gases or metabolites. Daily blood glucose (Figure 4A) and blood Ca^2+^ (Supplementary Figure S5) concentrations did not differ between IUGR lambs and controls, but glucose was less (*p* ≤ 0.05) and Ca^2+^ was greater (*p* ≤ 0.05) for IUGR+CLEN lambs than for controls. Plasma insulin concentrations were less (*p* ≤ 0.05) for IUGR and IUGR+CLEN lambs than for controls (Figure 4B). Glucose-to-insulin ratios were greater (*p* ≤ 0.05) for IUGR lambs than for controls and were intermediate for IUGR+CLEN lambs (Figure 4C). Blood lactate (0.60 ± 0.04 mM), HCO_3_^−^ (26.0 ± 0.3 mM), pO_2_ (79.4 ± 1.2 mmHg), base excess (2.6 ± 0.5 mM), K^+^ (4.22 ± 0.04 mM), Na^+^ (152.9 ± 0.9 mM), plasma triglycerides (Figure 5A), and plasma HDL-cholesterol (Figure 5B) concentrations did not differ among experimental groups. Blood plasma NEFA concentrations were greater (*p* ≤ 0.05) and BUN concentrations were less (*p* ≤ 0.05) for IUGR but not IUGR+CLEN lambs than for controls (Figures 5C, D, respectively). Blood pH was less (*p* ≤ 0.05) for IUGR and IUGR+CLEN lambs than for controls and less (*p* ≤ 0.05) for IUGR+CLEN than IUGR lambs (Figure 6A). Conversely, blood pCO_2_ was greater (*p* ≤ 0.05) for IUGR and IUGR+CLEN lambs compared to controls and greater (*p* ≤ 0.05) for IUGR+CLEN than for IUGR lambs (Figure 6B). Blood oxyhemoglobin concentrations were less (*p* ≤ 0.05) and carboxyhemoglobin concentrations were greater (*p* ≤ 0.05) for IUGR and IUGR+CLEN lambs compared to controls (Figures 6C, D, respectively). Blood carboxyhemoglobin concentrations were greater (*p* ≤ 0.05) and blood pH was less (*p* ≤ 0.05) for males than females. Blood glucose concentrations were greater (*p* ≤ 0.05) for singletons than for twin-born lambs.

### 3.5 Muscle β2 adrenoceptor, lipid droplet, and myoblast profiles

**FIGURE 7:**
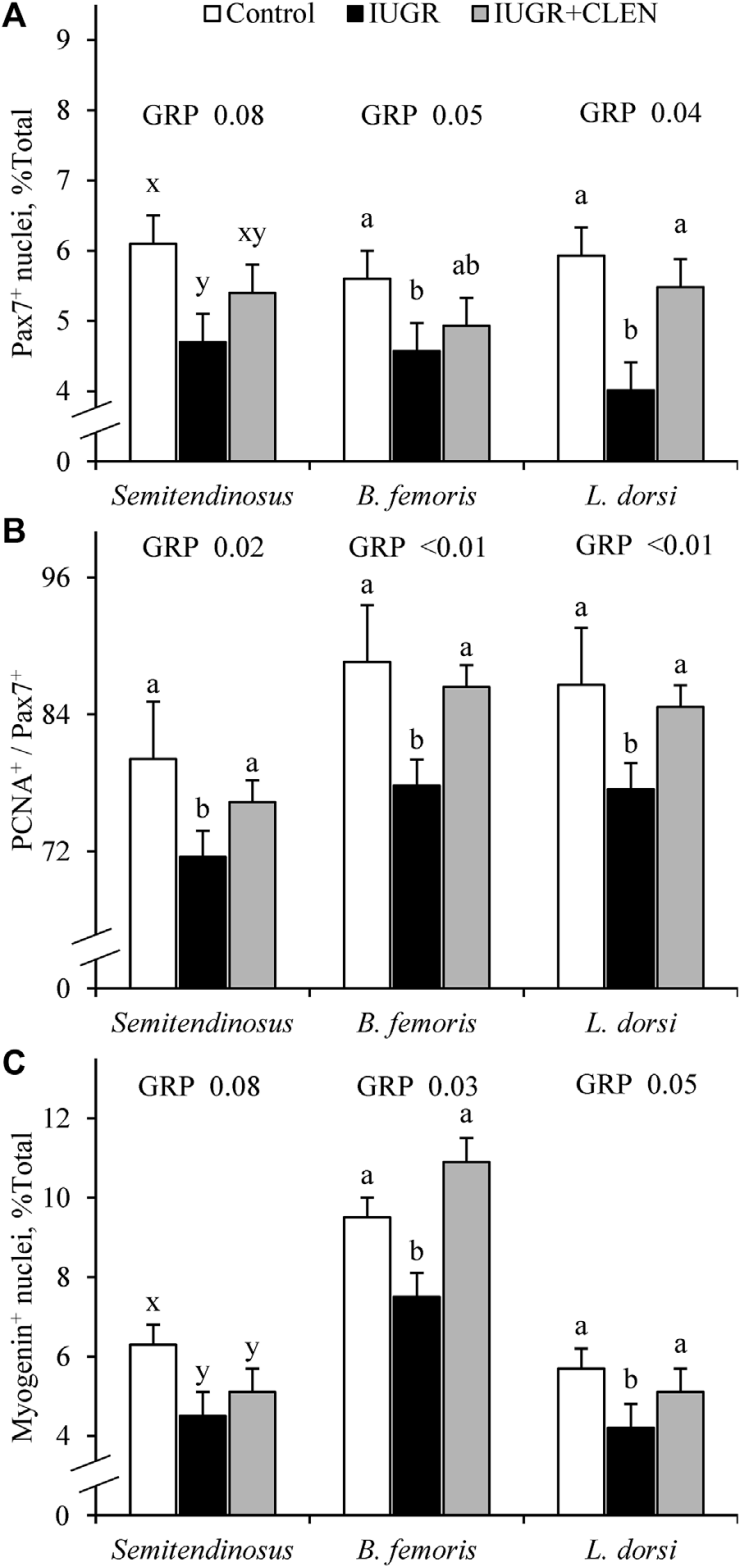
Myoblast profiles in skeletal muscle from IUGR-born lambs administered daily injectable clenbuterol. *Semitendinosus*, *biceps femoris*, and *longissimus dorsi* cross-sections were collected from controls (*n* = 13), IUGR lambs (*n* = 12), and IUGR+CLEN lambs (*n* = 11) at 60 days of age. Data are presented for percentages of nuclei expressing pax7 (i.e., myoblasts) **(A)**, percentages of pax7^+^ nuclei co-expressing PCNA (i.e., proliferating myoblasts) **(B)**, and percentages of nuclei expressing myogenin (i.e., differentiated myoblasts) **(C)** averaged from 800 nuclei across 3 non-overlapping fields of view. Effects of experimental group (GRP) were evaluated and noted where significant (*p* < 0.05) or tending toward significant (*p* < 0.10). ^a, b, c^ Means with different superscripts differ (*p* < 0.05). ^x, y, z^ Means with different superscripts tend to differ (*p* < 0.10).

**FIGURE 8:**
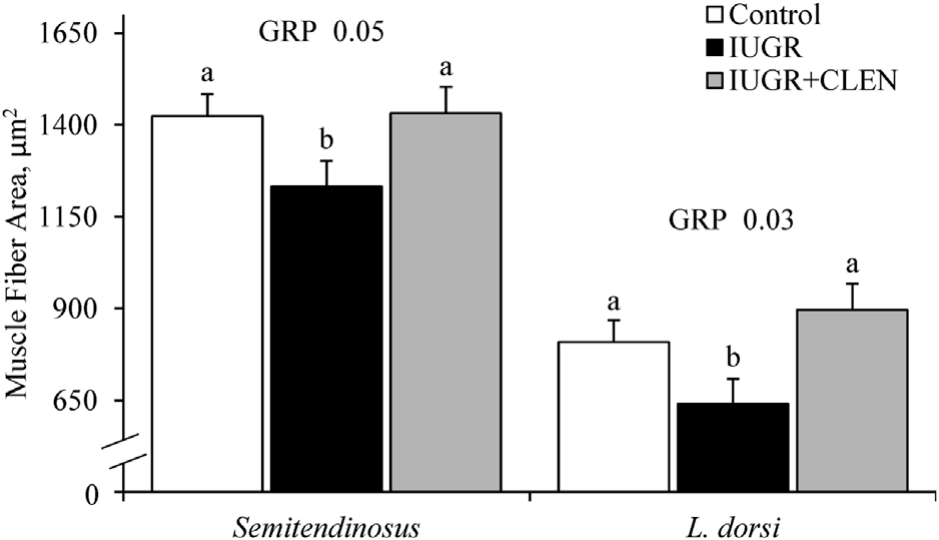
Myofiber size in skeletal muscle from IUGR-born lambs administered daily injectable clenbuterol. *Semitendinosus* and *longissimus dorsi* cross-sections were collected from controls (*n* = 13), IUGR lambs (*n* = 12), and IUGR+CLEN lambs (*n* = 11) at 60 days of age. Data are presented for mean fiber size averaged from a minimum of 100 fibers across 3 non-overlapping fields of view. Effects of experimental group (GRP) were evaluated and noted where significant (*p* < 0.05). ^a, b^ Means with different superscripts differ (*p* < 0.05).

**FIGURE 9:**
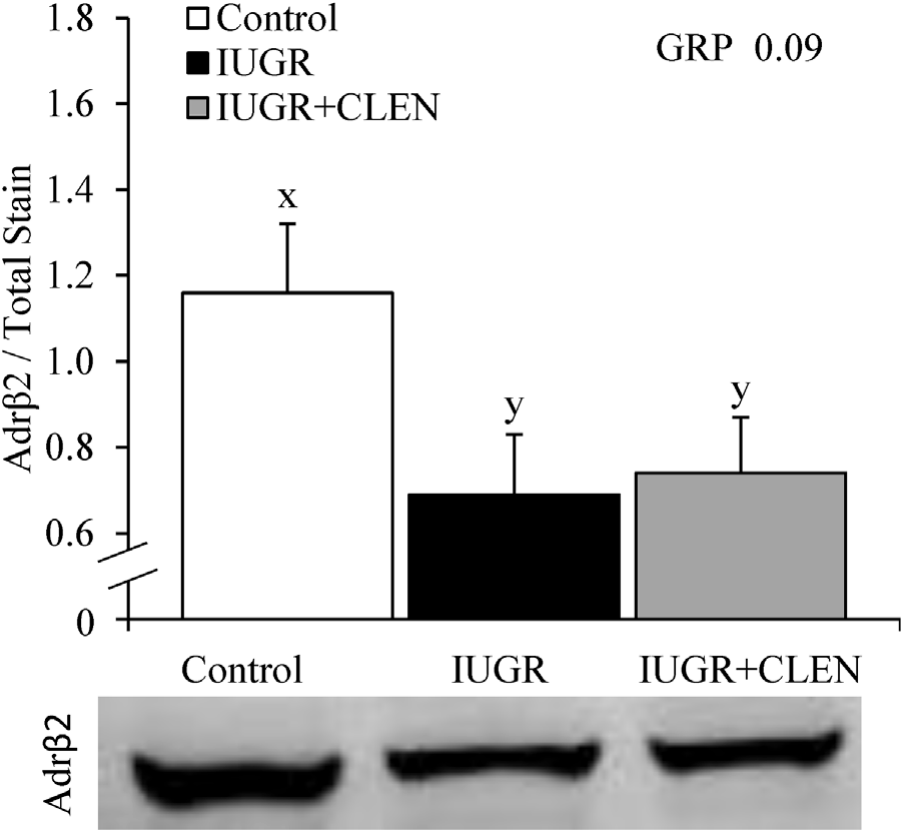
Skeletal muscle protein expression for IUGR-born lambs administered daily injectable clenbuterol. *Semitendinosus* muscle samples were collected from controls (*n* = 13), IUGR lambs (*n* = 12), and IUGR+CLEN lambs (*n* = 11) at 60 days of age. Data are presented for skeletal muscle β2 adrenoreceptor content determined by protein immunoblot. Effects of experimental group (GRP) were evaluated and noted where significant (*p* < 0.05) or tending towards significant (*p* < 0.10). ^x, y^ Means with different superscripts differ (*p* < 0.10). Representative gel micrographs are presented in the lower pane.

**FIGURE 10:**
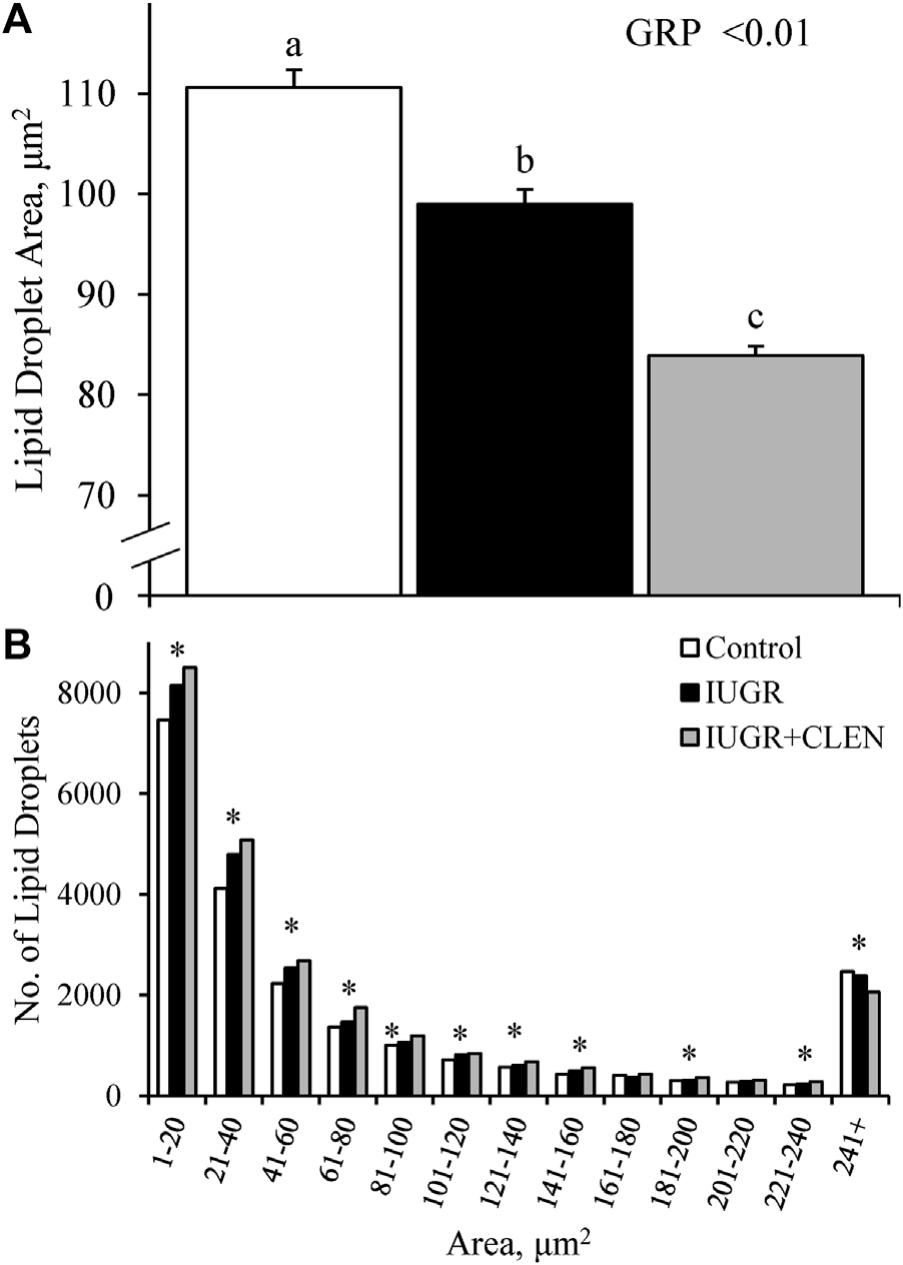
Skeletal muscle lipid droplet profiles for IUGR-born lambs administered daily injectable clenbuterol. Lipid droplets were assessed in cross-sections of *semitendinosus* collected at 60 days of age from controls (*n* = 13), IUGR lambs (*n* = 12), and IUGR+CLEN lambs (*n* = 11). Data are presented for average lipid droplet size **(A)** and lipid droplet size distribution **(B)**. Effects of experimental group (GRP) were evaluated and noted where significant (*p* < 0.05). ^a, b, c^ Means with different superscripts differ (*p* < 0.05). * Counts differ (*p* < 0.05) among experimental groups with the size category.

Representative images for protein immunohistochemistry are shown in Supplementary Figure S6. At necropsy, the percentages of pax7^+^ nuclei were less (*p* ≤ 0.05) in the *longissimus dorsi* and *biceps femoris* and tended to be less (*p* = 0.08) in the *semitendinosus* from IUGR but not IUGR+CLEN lambs compared to controls (Figure 7A). The percentages of pax7^+^/PCNA^+^ nuclei were less (*p* ≤ 0.05) for IUGR but not IUGR+CLEN lambs than for controls in all three muscles (Figure 7B). The percentages of myogenin^+^ nuclei tended to be less (*p* ≤ 0.05) in the *semitendinosus* from IUGR and IUGR+CLEN lambs than for controls (Figure 7C). The percentages of myogenin^+^ nuclei were less (*p* ≤ 0.05) in the *longissimus dorsi* and *biceps femoris* from IUGR but not IUGR+CLEN lambs compared to controls. Mean cross-sectional muscle fiber areas were smaller (*p* ≤ 0.05) in *semitendinosus* and *longissimus dorsi* from IUGR but not IUGR+CLEN lambs than for controls (Figure 8). We were unable to assess *biceps femoris* fiber area, as these samples were lost to a storage issue. *Semitendinosus* Adrβ2 protein concentrations tended to be less (*p* = 0.09) for IUGR and IUGR+CLEN lambs than for controls (Figure 9). Representative images are shown for lipid staining in Supplementary Figure S7. Lipid droplets in the *semitendinosus* were smaller (*p* ≤ 0.05) in average area for IUGR and IUGR+CLEN lambs compared to controls and were smaller (*p* ≤ 0.05) for IUGR+CLEN than for IUGR lambs (Figure 10A). Lipid droplets larger than 240 μm^2^ were fewer (*p* ≤ 0.05) in number and lipid droplets smaller than 240 μm^2^ were greater (*p* ≤ 0.05) in number for IUGR and IUGR+CLEN lambs than for controls (Figure 10B). No myogenic factors, muscle proteins, or muscle lipids assessed in this study differed between sexes or between singletons and twin-born lambs.

## 4 Discussion

In this study, we found that sustained postnatal β2 adrenergic stimulation improved poor muscle growth and body composition in lambs following chronic prenatal stress. Maternofetal heat stress during mid-gestation induced substantial IUGR, which caused lambs to be born smaller and to gain weight at a slower rate. Moreover, IUGR-born lambs exhibited asymmetric body composition, as morphometrics and carcass characteristics were consistent with greater relative fat deposition and reduced lean muscle growth. Reductions in muscle β2 adrenoreceptor content previously observed in IUGR-born lambs at 30 days of age (Cadaret et al., 2022) were present at 60 days of age in this study, reflecting the persistence of reduced β2 adrenergic regulatory tone. Despite this reduction in receptor content, daily administration of the β2 adrenergic agonist clenbuterol from birth to weaning increased indicators of lean muscle mass and reduced indicators of adiposity in IUGR-born juvenile lambs. In addition to resolving IUGR-induced deficits in muscle protein content, fat-to-protein ratio, and loin-eye area, β2 adrenergic stimulation also improved indicators of reduced myoblast function across multiple muscles, which in turn coincided with larger muscle fiber sizes. Together, these findings demonstrate that growth and body composition disparities in IUGR-born offspring coincide with adaptive fetal programming that diminishes skeletal muscle regulation by β2 adrenergic pathways. Moreover, improved muscle growth, lean mass indicators, and body composition observed when β2 adrenergic activity was exogenously stimulated indicate that β2 adrenergic deficits contribute in part to poor growth outcomes. As such, targeting β2 adrenergic pathways may offer a strategy to recover efficient muscle growth and body symmetry in animals born with low birthweight following heat stress-induced IUGR. It is also important to note that tissue regulation by the β2 adrenergic system is robust, and the possibility of unidentified secondary effects of exogenous stimulation on muscle growth and metabolism cannot be discounted.

Reduced β2 adrenergic regulation of skeletal muscle appeared in concert with impaired muscle growth capacity and consequent asymmetric body composition that characterized IUGR-born lambs. As juveniles, these lambs consistently exhibited reductions of 20%–30% across multiple indicators of muscle mass assessed in both the live animal and the carcass. By comparison, IUGR bodyweights at necropsy were reduced by only 15%, body length and crown circumference by less than 5%, and cannon bone length and brain weights were not reduced. This disproportionate restriction of muscle mass is an IUGR hallmark that arises from fetal programming adaptions to chronic periods of low fetal nutrient and O_2_ availability (Bell and Greenwood, 2016; Soto et al., 2017; Rozance et al., 2018). Nutrient repartitioning to preserve brain, bone, and endocrine tissues increases fetal survival of low-nutrient conditions but also produces asymmetry of fetal growth (Brown, 2014; Posont and Yates, 2019). The more profound impairment of muscle growth relative to brain and skeletal growth persisted well after birth in this study. This confirms that asymmetric growth observed in IUGR fetuses and newborns (Posont et al., 2021; Cadaret et al., 2022) is the product of tissue programming. We can presume that muscle is a primary target of nutrient repartitioning due to its high consumption of glucose, which has been estimated at ∼60% of the body’s total glucose utilization and ∼85% of the body’s insulin-stimulated utilization (DeFronzo et al., 1981). Slower muscle growth in IUGR fetuses is facilitated in part by intrinsic deficits in myoblast function (Yates et al., 2014; Brown and Hay, 2016; Soto et al., 2017; Posont et al., 2022) that restrict myonuclear accumulation by muscle fibers (Yates et al., 2016; Cadaret et al., 2019), a rate-limiting step for postnatal hypertrophic muscle growth (Allen and Rankin, 1990; Wilson et al., 1992). Our present findings of diminished proliferating and differentiated myoblast populations in IUGR juvenile loin and hindlimb muscles demonstrate that functional deficits in fetal myoblasts likewise persist in offspring. This establishes myoblast programming as a key mechanistic link between prenatal stress and lifelong deficits in muscle growth capacity. Recent studies also found that IUGR fetal muscle synthesizes protein at reduced rates, which further slows muscle growth (Rozance et al., 2018; Cilvik et al., 2021). Our study revealed a modest reduction of IUGR muscle protein content that was presumably a result of those previously-identified deficits in protein synthesis capacity. Impaired myoblast functional capacity and protein synthesis are clear impediments to postnatal hypertrophic muscle growth. Indeed, we found that IUGR-born lambs exhibited ∼20% smaller muscle fibers at weaning age, which in turn contributed to smaller loin-eye areas, lighter *flexor digitorum superficialis* muscles, and smaller hindlimbs. Diminished muscle mass negatively impacts whole-body glucose homeostasis in IUGR fetuses and offspring (Brown, 2014; Yates et al., 2018; Gibbs and Yates, 2021). When nutrient availability is restored after birth, IUGR programming mechanisms for thrifty nutrient utilization lead to greater deposition of adipose tissue (Desai and Ross, 2011). In fact, bodyweight deficits are often resolved in IUGR-born offspring by adiposity-driven catch-up growth that disproportionately increases body fat and contributes to hallmark asymmetric body composition (Desai and Ross, 2011; Zinkhan et al., 2018). In the present study, greater adiposity was reflected in body composition and carcass analyses, which revealed that IUGR-born lambs exhibited small reductions in total fat mass but greater percentages of fat in soft tissues. Our findings also indicate that this juvenile stage (i.e., around the traditional time of weaning) is when increased adiposity becomes apparent in IUGR offspring, as studies found normal or even reduced adiposity at earlier ages (Duffield et al., 2009; Gatford et al., 2013; Wallace et al., 2020) and greater adiposity at later ages (Wallace et al., 2018).

Postnatal stimulation of β2-specific adrenergic activity improved much of the deficient muscle growth and body composition observed in IUGR-born juvenile lambs. This demonstrates that the ability of β agonists to increase muscle mass and leanness, which is well documented in uncompromised animals and people (Gibbs and Yates, 2021; Hostrup and Onslev, 2022), can be leveraged to overcome stress-altered development. Indeed, diminished muscle Adrβ2 content in this and other studies of IUGR (Yates et al., 2012; Yates et al., 2019; Cadaret et al., 2022) reflects impairment of this key pathway for muscle growth. Yet, sustained stimulation of β2 adrenergic activity in the present study was particularly effective in recovering indicators of hypertrophic muscle growth, including myoblast function, muscle fiber size and protein content, and fat mass. The stimulatory effects resulted in better lean mass indicators and body symmetry, despite the reduced receptor expression. Fasted bodyweights were not recovered by clenbuterol treatment even with greater apparent muscle growth, which was comparable to findings in normal lambs treated with β2 adrenergic agonists (Beermann et al., 1987; Beermann, 2002). Because clenbuterol did not improve reduced muscle Adrβ2 content, we presume that the underlying programming was not resolved but rather mitigated. Importantly, Adrβ2 content was also not further reduced by daily treatment with the β2 adrenergic agonist.

Sustained stimulation of β2 adrenergic activity improved some but not all indicators of metabolic deficits in IUGR-born lambs. Much like IUGR neonates in a previous study (Yates et al., 2019), IUGR-born juvenile lambs in the present study exhibited less circulating insulin concentrations at comparable resting glycemia. This may have been due to transient increases in peripheral insulin sensitivity for glucose deposition or to permanent β cell dysfunction, both of which have been observed in IUGR-born offspring at earlier ages (Camacho et al., 2017; Yates et al., 2019; Cadaret et al., 2022). Pancreatic islet dysfunction was previously linked to chronic prenatal exposure to high circulating catecholamines, which suppressed islet function via α2 adrenoceptors (Macko et al., 2013). Sustained adrenergic stimulation of fetal islets causes a transient increase in sensitivity to glucose (Leos et al., 2010; Chen et al., 2014; Chen et al., 2017; Kelly et al., 2018) that ultimately gives way to fewer β cells, reduced insulin content, and diminished insulin secretion (Limesand et al., 2006; Kelly et al., 2018). In IUGR fetal sheep, islets developed an almost 80% reduction in β cell mass near term (Boehmer et al., 2017; Limesand and Rozance, 2017). Thus, it is reasonable to conclude that reduced circulating insulin at resting glycemia in our IUGR-born lambs reflected islet dysfunction. Despite the supportive role of β2 adrenergic activity in fetal islet development (Borden et al., 2013; Ceasrine et al., 2018), postnatal β2 adrenergic stimulation did not recover resting blood insulin concentrations or glucose-to-insulin ratios. This indicates that greater β2 adrenergic activity was not sufficient to overcome developmental deficits in pancreatic islets.

Hyperlipidemia was present in IUGR-born lambs, which coincided with greater skeletal muscle fat content and indicators of higher body fat percentages. Elevated circulating free fatty acids in our IUGR-born juveniles were consistent with studies at younger ages (Alvino et al., 2008; Drake et al., 2022) and resembled lipid profiles in IUGR-born pigs, humans, and rodents in adulthood (Malo et al., 2013; Dunlop et al., 2015; Shen et al., 2018). Hyperlipidemia, together with greater intramuscular lipid accumulation but not subcutaneous fat deposition, may be indicative of impaired fatty acid metabolism. Recent studies found that lipid accumulation in non-adipose tissues like skeletal muscle may result from mitochondrial dysfunction that reduces fatty acid oxidation capacity (Dunlop et al., 2015; Drake et al., 2022). Interestingly, circulating triglycerides were not elevated in our IUGR-born lambs, although it is reasonable to speculate that the greater skeletal muscle lipid content may have included increased intramuscular triglycerides, as previously reported in IUGR-born pigs (Li et al., 2015), and 8-month old IUGR-born sheep (Sandoval et al., 2020). The hyperlipidemic phenotype has been directly linked to insulin resistance and progressively worsening metabolic dysfunction in IUGR-born offspring (Dunlop et al., 2015; Drake et al., 2022). However, early-life β2 adrenergic stimulation appeared to rescue lipid homeostasis in our IUGR-born lambs. Along with reducing fat-to-protein indicators and back-fat thickness, treatment with clenbuterol reduced circulating free fatty acids, HDL-cholesterol, and triglycerides, highlighting its ability to upregulate fatty acid clearance and metabolism (Byrem et al., 1998; Hostrup and Onslev, 2022). Despite previous evidence for the role of inflammatory programming (Hicks and Yates, 2021), greater circulating NEFA in these IUGR lambs did not correspond to changes in circulating immune cell populations or hematological indicators, which would have been consistent with fat-induced inflammation. Stimulating β2 adrenergic activity also improved indicators of protein homeostasis. Our IUGR-born juveniles exhibited reduced circulating blood urea nitrogen, which parallels findings in the IUGR fetus (Brown and Hay, 2016; Rozance et al., 2018) and indicates dysregulation of protein cycling. Impaired protein synthesis/catabolism balance would be consistent with the reduced muscle size and whole-body lean mass observed in the present study. However, increased β2 adrenergic activity recovered circulating blood urea nitrogen, which previous findings indicate were likely the result of Adrβ2 pathways engaging the Akt-mTORC1 cascade that upregulates protein synthesis (Posont et al., 2017; Chia et al., 2019).

A small number of growth and metabolic outputs that were not influenced by the IUGR condition were responsive to β2 adrenergic stimulation, which was expected. For example, reduced circulating concentrations of glucose and triglycerides were consistent with the known effects of β2 adrenergic agonists (Konstandi et al., 2019; Kalinovich et al., 2020; van Beek et al., 2023). Likewise, the modest increase in circulating Ca^2+^ concentrations, greater loin eye area, and reduced backfat thickness were also consistent with outcomes of supplementing β2 agonists as growth promotors in uncompromised animals (Muir, 1988; Bell et al., 1998; Byrem et al., 1998). Although it is important to understand potential off-target effects, these findings are further evidence that IUGR muscle retained meaningful adrenergic responsiveness, which was not certain due to the previously-documented reduction in Adrβ2 content (Cadaret et al., 2022).

From this study, we can conclude that reduced β2 adrenergic activity was partially responsible for deficits in muscle growth, body composition, and metabolic homeostasis observed in IUGR-born juvenile lambs. Moreover, stimulating β2 adrenergic activity from birth to weaning improved several well-characterized muscle-centric outcomes of IUGR, despite a persistent reduction in muscle β2 adrenoreceptor content. Notable improvements in muscle growth and body composition indicated that tissues remained responsive to β2 stimulatory activity, and thus postnatal adrenergic manipulation may provide a strategy to recover growth efficiency and metabolic outcomes of animals born with low birthweight due to IUGR. These findings provide the fundamental basis for future studies aimed at developing practical supplementation strategies for improving animal welfare, productivity, and value heat stress-induced low birthweight livestock.

## Data Availability

The raw data supporting the conclusions of this article will be made available by the authors, without undue reservation.
