# Supplementary material for: Daily injection of the β2 adrenergic agonist clenbuterol improved poor muscle growth and body composition in lambs following heat stress-induced intrauterine growth restriction
Source: Front Physiol. 2023 Sep 6;14:1252508. doi: 10.3389/fphys.2023.1252508 (PMC10516562; doi:10.3389/fphys.2023.1252508)
Supplement: Supplementary file 1 [file DataSheet1.docx]

*Daily injection of the β2 adrenergic agonist clenbuterol improved poor muscle growth and body composition in lambs following heat stress-induced intrauterine growth restriction.*

# Supplementary Figures and Tables

## Supplementary Figures


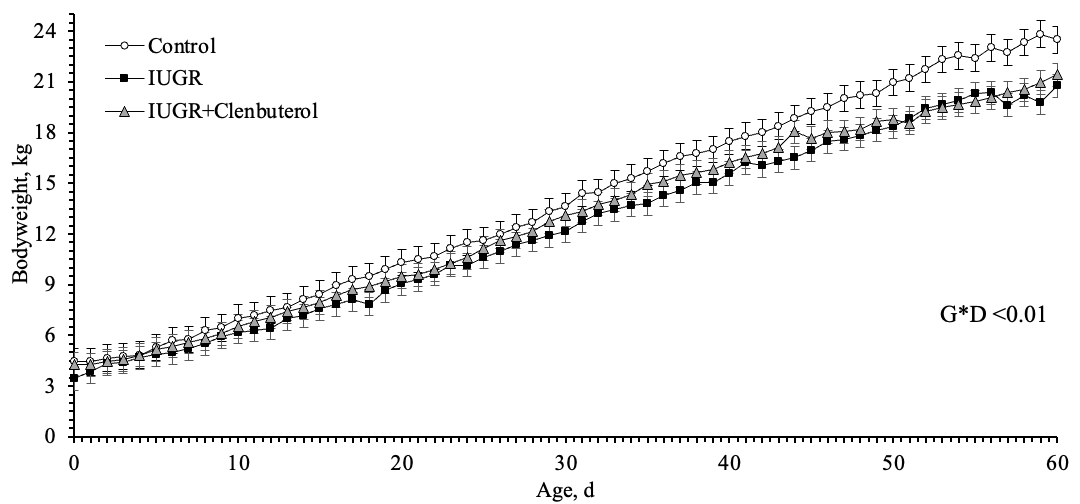


**Supplementary Figure 1.** Growth of IUGR-born lambs administered daily injectable clenbuterol. Daily bodyweight was assessed in controls (n = 13), IUGR lambs (n = 12), and IUGR+CLEN lambs (n = 11) from birth to 60 d of age. Effects of experimental group (GRP), day (DAY), and the interaction (G*D) were evaluated and noted where significant (*P* < 0.05). Bodyweights were less (*P* < 0.05) for IUGR and IUGR+CLEN lambs than controls on all days.

**
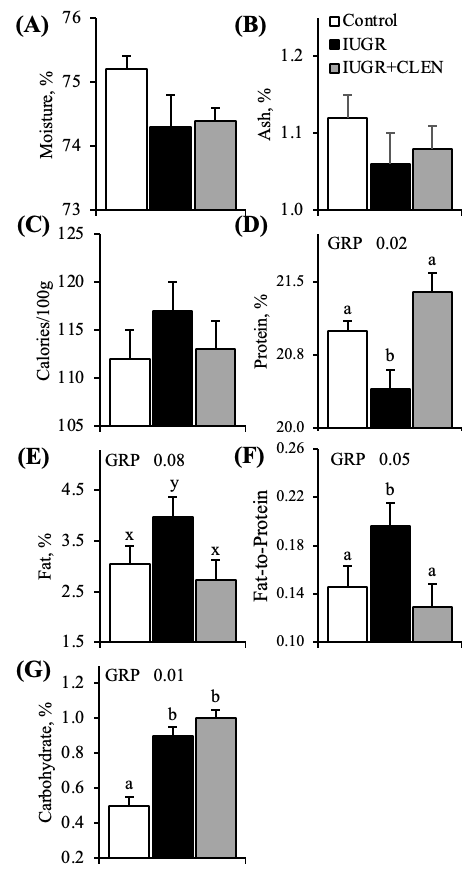
**

**Supplementary Figure 2.** Loin muscle composition for IUGR-born lambs administered daily injectable clenbuterol. Proximate analysis was performed on *longissimus dorsi* samples collected at 60 d of age from controls (n = 13), IUGR lambs (n = 12), and IUGR+CLEN lambs (n = 11). Data are presented for moisture content (a.), ash content (b.), calorie content (c.), protein content (d.), fat content (e.), fat-to-protein ratios (f.), and carbohydrate content (g.). Effects of experimental group (GRP) were evaluated and noted where significant (*P* < 0.05) or tending toward significant (*P* < 0.10). ^a, b^ Means with different superscripts differ (*P* < 0.05). ^x, y^ Means with different superscripts tend to differ (*P* < 0.10).

**
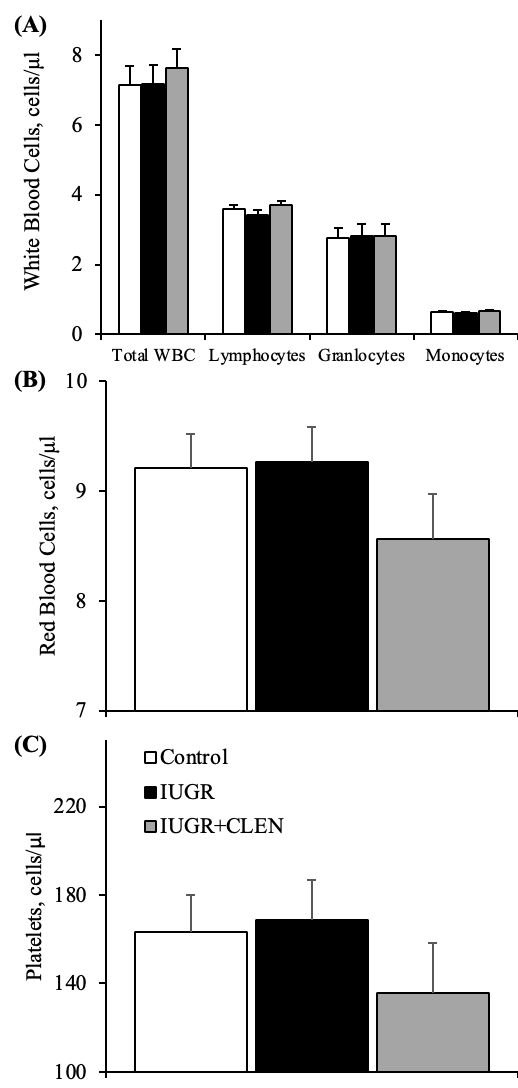
**

**Supplementary Figure 3.** Daily blood cell profiles for IUGR-born lambs administered daily injectable clenbuterol. Arterial blood samples were collected from controls (n = 13), IUGR lambs (n = 12), and IUGR+CLEN lambs (n = 11) from the 55^th^ to 50^th^ d of age. Data are presented for concentrations of total and differential white blood cells (a.), red blood cells (b.), and platelets (c.). Effects of experimental group (GRP), day (DAY), and the interaction (G*D) were evaluated but were not different.


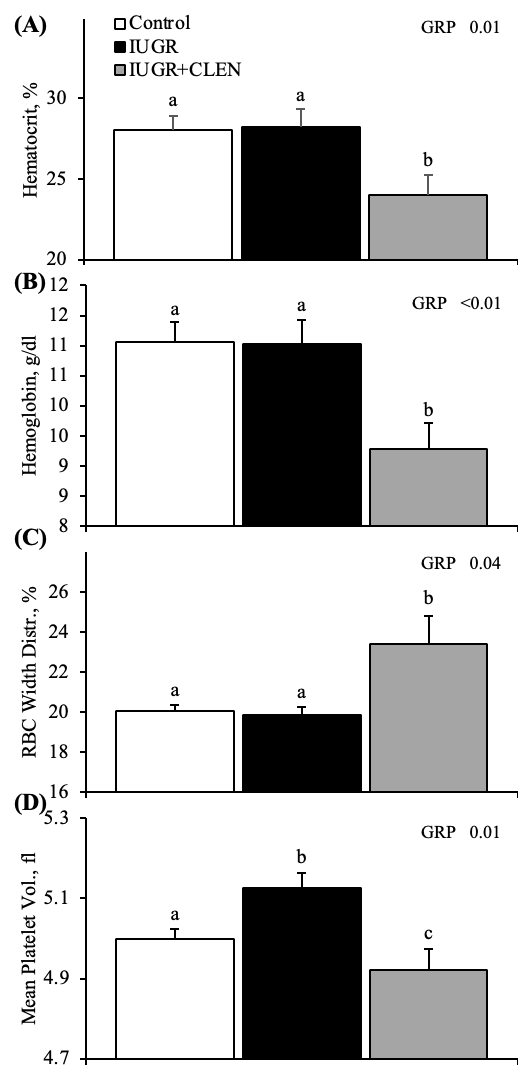


**Supplementary Figure 4.** Daily hematology for IUGR-born lambs administered daily injectable clenbuterol. Arterial blood samples were collected from controls (n = 13), IUGR lambs (n = 12), and IUGR+CLEN lambs (n = 11) from the 55^th^ to 50^th^ d of age. Data are presented for hematocrit (a.), hemoglobin (b.), red blood cell distribution width (c.), and mean platelet volume (d.). Effects of experimental group (GRP), day (DAY), and the interaction (G*D) were evaluated and noted where significant (*P* < 0.05). ^a, b, c^ Means with different superscripts differ (*P* < 0.05).


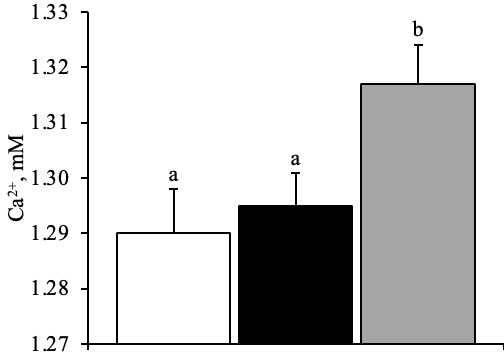


**Supplementary Figure 5.** Daily blood Ca^2+^ concentrations for IUGR-born lambs administered daily injectable clenbuterol. Arterial blood samples were collected from controls (n = 13), IUGR lambs (n = 12), and IUGR+CLEN lambs (n = 11) from the 55^th^ to 50^th^ d of age. Effects of experimental group (GRP), day (DAY), and the interaction (G*D) were evaluated and noted where significant (*P* < 0.05). ^a, b^ Means with different superscripts differ (*P* < 0.05).


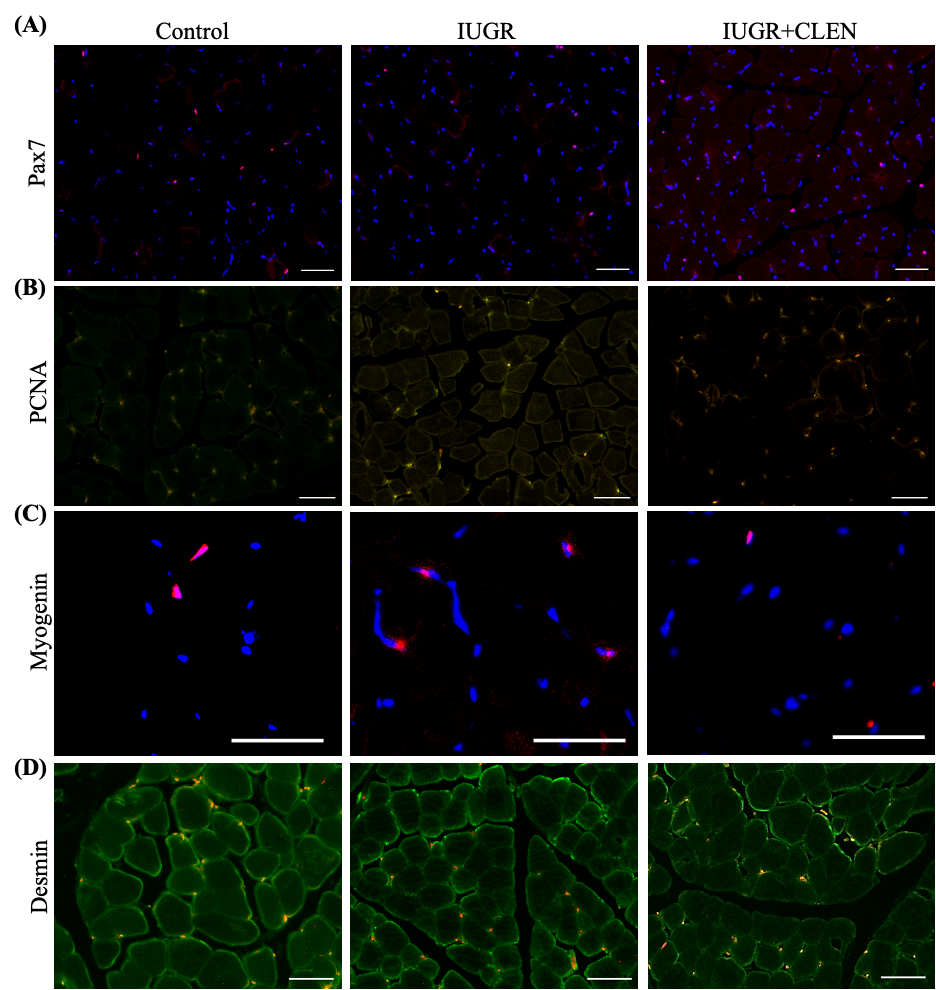


**Supplementary Figure 6.** Representative micrographic images for skeletal muscle immunohistochemistry. (a.) Total myoblasts were estimated from pax7^+^ (red) percentages of total nuclei (DAPI^+^; blue). (b.) Proliferating myoblasts were estimated from percentages of pax7^+^ (red) nuclei co-expressing proliferating-cell nuclear antigen (PCNA^+^, green). (c.) Differentiated myoblasts were estimated from myogenin^+^ (red) percentages of total nuclei (DAPI^+^; blue). (d.) Average muscle fiber cross-sectional areas were estimated from desmin^+^ (green) tissues. Scale bar = 50 μm.


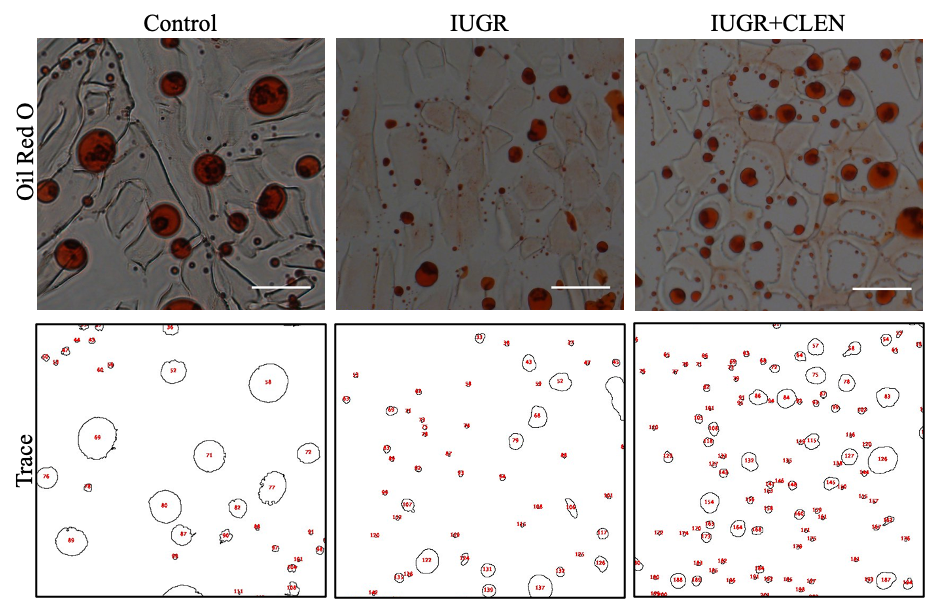


**Supplementary Figure 7.** Representative micrographic images for skeletal muscle lipid staining. The top row shows lipid droplets identified by Oil Red O total lipid staining. The lower row shows the software-generated measurements of the lipid droplets in the above images. Scale bar = 50 μm.

## Supplementary Tables

| **Supplemental Table 1.** Nutrient analysis of diet fed to all lambs beginning at 30 d of age. | | |
| --- | --- | --- |
| Component |  | Amount |
| Crude Protein^1^, % (Minimum) |  | 36.0 |
| Crude Fat, % (Minimum) |  | 1.0 |
| Crude Fiber, % (Maximum) |  | 11.5 |
| Ca^2+^, % ( Minimum) |  | 4.5 |
| Ca^2+^, % ( Maximum) |  | 5.5 |
| P, % ( Minimum) |  | 0.85 |
| NaCl, % ( Minimum ) |  | 2.5 |
| NaCl, % ( Maximum) |  | 3.5 |
| Se, ppm (Minimum) |  | 1.3 |
| Vitamin A, IU/kg (Minimum) |  | 909 |
| Lasalocid Sodium^2^, g/ton |  | 136 |
| ^1^ Includes not more than 4% equivalent crude protein from non-protein nitrogen.  ^2^ Active ingredient. | | |

| **Supplemental Table 2.** Absolute and relative growth measurements at 60 d of age in IUGR-born lambs administered daily injectable clenbuterol. | | | | | |
| --- | --- | --- | --- | --- | --- |
|  | Experimental Group | | |  | *P-*value |
|  | Control | IUGR | IUGR+CLEN^1^ |  |  |
| Metric, cm |  |  |  |  |  |
| Crown Circumference | 37.7±0.50^a^ | 36.1±0.40^b^ | 36.5±0.40^b^ |  | < 0.001 |
| Abdominal Circumference | 58.2±1.00^a^ | 55.9±1.00^b^ | 56.1±1.00^b^ |  | < 0.001 |
| Crown-Rump Length | 83.6±0.70^a^ | 79.8±0.60^b^ | 80.4±0.70^b^ |  | 0.01 |
| Cannon Bone | 15.0±0.10 | 14.7±0.1 | 14.7±0.1 |  | NS |
| Metric/BW, cm/kg |  |  |  |  |  |
| Crown Circumference | 3.59±0.05^a^ | 4.18±0.19^b^ | 3.95±0.11^b^ |  | < 0.001 |
| Abdominal Circumference | 5.34±0.08^a^ | 6.04±0.18^b^ | 5.79±0.14^b^ |  | < 0.001 |
| Crown-Rump Length | 7.75±0.15^a^ | 8.77±0.33^b^ | 8.39±0.23^b^ |  | 0.001 |
| Cannon Bone | 1.45±0.05^a^ | 1.70±0.08^b^ | 1.61±0.06^b^ |  | < 0.001 |
| Metric/BL, cm/cm |  |  |  |  |  |
| Crown Circumference | 0.455±0.004 | 0.461±0.003 | 0.461±0.003 |  | NS |
| Abdominal Circumference | 0.695±0.006 | 0.701±0.006 | 0.697±0.006 |  | NS |
| Cannon Bone | 0.182±0.003^a^ | 0.188±0.003^b^ | 0.186±0.003^b^ |  | 0.01 |
| Cannon Bone/ Abdominal Circumference, cm/cm | 0.263±0.005^x^ | 0.270±0.005^y^ | 0.268±0.005^y^ |  | 0.06 |
| Values are expressed as ls means ± standard error.  ^a, b^ Means with different superscripts differ (*P* ≤ 0.05).  ^x, y^ Means with different superscripts tend to differ (*P* ≤ 0.10).  ^1^ Daily treatment with 0.8 μg/kg injectable (IM) clenbuterol HCl.  BL, body length (i.e., crown-rump length); BW, bodyweight; IUGR, intrauterine growth restriction; NS, not significant. | | | | | |
